# Magnesium Sensing Regulates Intestinal Colonization of Enterohemorrhagic Escherichia coli O157:H7

**DOI:** 10.1128/mBio.02470-20

**Published:** 2020-11-10

**Authors:** Yutao Liu, Runhua Han, Junyue Wang, Pan Yang, Fang Wang, Bin Yang

**Affiliations:** a TEDA Institute of Biological Sciences and Biotechnology, Nankai University, TEDA, Tianjin, People’s Republic of China; b The Key Laboratory of Molecular Microbiology and Technology, Ministry of Education, Tianjin, People’s Republic of China; University of Pittsburgh

**Keywords:** bacterial adherence, magnesium, locus of enterocyte effacement (LEE), virulence, Z4267, gene regulation

## Abstract

The large intestinal pathogen enterohemorrhagic Escherichia coli (EHEC) O157:H7 detects host cues to regulate virulence gene expression during colonization and infection. However, virulence regulatory mechanisms of EHEC O157:H7 in the human large intestine are not fully understood. Herein, we identified a virulence-regulating pathway where the PhoQ/PhoP two-component regulatory system senses low magnesium levels and signals to the O island 119-encoded Z4267 (LmiA; low magnesium-induced regulator A), directly activating loci of enterocyte effacement genes to promote EHEC O157:H7 adherence in the large intestine. Disruption of this pathway significantly decreased EHEC O157:H7 adherence in the mouse intestinal tract. Moreover, feeding mice a magnesium-rich diet significantly reduced EHEC O157:H7 adherence *in vivo*. This LmiA-mediated virulence regulatory pathway is also conserved among several EHEC and enteropathogenic E. coli serotypes; therefore, our findings support the use of magnesium as a dietary supplement and provide greater insights into the dietary cues that can prevent enteric infections.

## Abstract (precis)

Sensing specific gut metabolites is an important strategy for inducing crucial virulence programs by enterohemorrhagic Escherichia coli (EHEC) O157:H7 during colonization and infection. Here, we identified a virulence-regulating pathway wherein the PhoQ/PhoP two-component regulatory system signals to the O island 119-encoded low magnesium-induced regulator A (LmiA), which, in turn, activates locus of enterocyte effacement (LEE) genes to promote EHEC O157:H7 adherence in the low-magnesium conditions of the large intestine. This regulatory pathway is widely present in a range of EHEC and enteropathogenic E. coli (EPEC) serotypes. Disruption of this pathway significantly decreased EHEC O157:H7 adherence in the mouse intestinal tract. Moreover, mice fed a magnesium-rich diet showed significantly reduced EHEC O157:H7 adherence *in vivo*, indicating that magnesium may help in preventing EHEC and EPEC infection in humans.

## INTRODUCTION

Enterohemorrhagic Escherichia coli (EHEC) serotype O157:H7 (O157) is a clinically important food and waterborne pathogen that colonizes the human large intestine, causing diseases (1). Infection with EHEC O157 produces a wide spectrum of clinical manifestations, including asymptomatic infection, mild diarrhea, or severe diseases such as hemorrhagic colitis (HC) and hemolytic uremic syndrome (HUS) (2). One important characteristic of EHEC O157 virulence is its ability to form attaching and effacing (A/E) lesions on epithelial cells (2, 3). A/E lesions are characterized by bacterial attachment to host epithelium, followed by the induction of extensive actin rearrangements within the epithelial cells, culminating in the formation of pedestal-like structures underneath the bacteria (3). The ability to form A/E lesions is conferred by the locus of enterocyte effacement (LEE) pathogenicity island that is conserved among all A/E pathogens, including EHEC, enteropathogenic E. coli (EPEC), and Citrobacter rodentium (4, 5). LEE genes are mainly organized in five polycistronic operons (LEE1 to LEE5), where LEE1, LEE2, and LEE3 encode a type III secretion system (TTSS) that exports effecter molecules, LEE5 encodes intimin and its receptor Tir, which are necessary for intimate adherence to the host epithelial cells, and LEE4 encodes additional TTSS structural components, translocators, and effector proteins (4, 5).

LEE genes are subjected to strict regulation that ensures expression only under optimal environmental conditions while also avoiding intense metabolic cost and/or alerting the host immune system to ensure successful colonization (6). Several environmental signals present in the human intestinal tract have been demonstrated to affect EHEC O157 adherence or LEE gene expression; for example, the ability of EHEC O157 to adhere to epithelial cells or express LEE genes is enhanced in response to low pH, butyrate, ethanolamine, and fucose and is repressed by biotin, acyl-homoserine lactones, and d-serine (7). However, the precise molecular mechanisms underlying the regulation of these environmental signals and their effect on EHEC O157 virulence are not fully understood.

Magnesium plays several essential roles in all living cells, such as stabilizing membranes and ribosomes, neutralizing nucleic acids, and acting as a cofactor in a variety of enzymatic reactions (8). Humans can only obtain magnesium from external sources through gastrointestinal absorption (9). The main site of magnesium absorption is the small intestine, while no absorption occurs in the large intestine, indicating low magnesium levels in the human large intestine (9, 10). In bacteria, the PhoQ/PhoP two-component regulatory system responds to magnesium as its primary signal (11). Under low-magnesium conditions, PhoQ promotes the phosphorylated state of PhoP (PhoP-P), which, in turn, promotes transcription of magnesium transporter genes such as *mgtA* and *mgtB* (12). On the other hand, when magnesium levels are high, PhoQ favors the unphosphorylated state of PhoP (12). The PhoQ/PhoP two-component regulatory system is also required for the virulence of a variety of bacterial pathogens, including Salmonella enterica serovar Typhimurium (13), Yersinia pestis (14), Shigella flexneri (15), and Pseudomonas aeruginosa (16). However, involvement of the PhoP/PhoQ two-component regulatory system and its response signals in O157 virulence regulation has not been reported.

Genetic analyses showed that the EHEC O157 genome contains 177 genomic islands, termed O islands (OIs), that are not present in E. coli K-12 (17). Among these OIs, eight were found to be associated with EHEC O157 virulence. OI-148 contains the LEE pathogenicity island, and OI-45 and OI-93 encode Shiga toxins, which are implicated in severe human diseases such as HUS. OI-15 contains the AidA15 adhesin gene, which promotes EHEC O157 adherence both *in vitro* and *in vivo* (18). OI-48 encodes urease and the adhesin Iha, promoting pathogen adherence to the host intestinal epithelium (19). OI-57 harbors the putative virulence gene *adfO*, which encodes a factor that enhances the adherence capacity of EHEC O157 (20), and OI-71 encodes the non-LEE-encoded T3SS effector, NleA (21). OI-122 carries the virulence gene *efa1-lifA* encoding the EHEC adherence/lymphocyte inhibitory factor, which is required for *in vitro* adhesion of EHEC O157 to epithelial cells and inhibition of the host immune response (22). The majority of OI genes have not been assigned a function, however, and their effect on EHEC O157 virulence is still largely unknown.

In this study, we found that a novel virulence regulator, Z4267 (named LmiA for low magnesium-induced regulator A), encoded within OI-119, directly binds to the *ler* promoter region and activates transcription of *ler* (encoding a master regulator of LEE genes) under low-magnesium conditions. The response of LmiA to the low magnesium signal is mediated by the PhoQ/PhoP two-component system. Thus, we uncovered a novel virulence-regulating pathway—with OI-119-encoded LmiA as an essential element—that activates LEE genes to facilitate EHEC O157 colonization in the low-magnesium human large intestine.

## RESULTS

### OI-119 is required for EHEC O157 adherence capacity and LEE gene expression.

**FIG 1:**
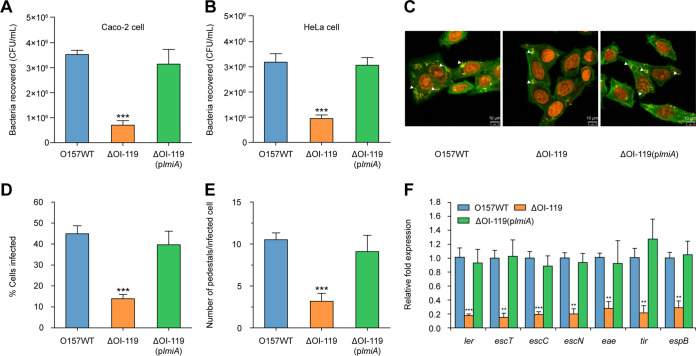
OI-119 is required for EHEC O157 adherence capacity and LEE gene expression. (A) Adherence capacity of O157 WT, ΔOI-119, and ΔOI-119(p*_lmiA_*) to Caco-2 cells. (B) Adherence capacity of O157 WT, ΔOI-119, and ΔOI-119(p*_lmiA_*) to HeLa cells. (C) FAS assay of HeLa cells infected with O157 WT, ΔOI-119, and ΔOI-119(p*_lmiA_*). HeLa nuclei and bacteria were stained with propidium iodide (red), and HeLa cell actin cytoskeleton was stained with FITC-phalloidin (green). Pedestals are observed as green punctate structures associated with bacterial cells and are indicated by arrowheads. Scale bar, 10 μm. (D and E) FAS assay quantification of the percentage of HeLa cells infected (D) and the number of pedestals per infected cell (E). *n *= 300 cells per strain. (F) qRT-PCR of LEE gene expression in O157 WT, ΔOI-119, and ΔOI-119(p*_lmiA_*). O157 WT, EHEC O157 wild-type strain; ΔOI-119, OI-119 mutant; ΔOI-119(p*_lmiA_*), OI-119 mutant complemented with *lmiA*. Data are presented as the means ± SD; *n* = 3. **, *P ≤ *0.01; ***, *P ≤ *0.001 (Student's *t* test).

Our preliminary comparative transcriptome analysis indicated that genes within OI-119 and the LEE pathogenicity island were significantly downregulated 3 h after incubation of HeLa cells with EHEC O157 (23), which was further confirmed with quantitative reverse transcription PCR (qRT-PCR) in this study (Table S1). To further investigate whether OI-119 is associated with EHEC O157 virulence, we constructed a ΔOI-119 mutant and determined its adherence capacity. As shown in Fig. 1A, the ΔOI-119 mutant adhered to Caco-2 cells at a much lower level—approximately one-quarter of that of the EHEC O157 wild-type (WT) strain; the same result was also obtained when HeLa epithelial cells were used instead of Caco-2 cells (Fig. 1B). Both the WT and ΔOI-119 mutant exhibited similar growth rates *in vitro* (Fig. S1A), indicating that the decreased ability of the ΔOI-119 mutant to adhere to host epithelial cells was not due to a growth defect. Fluorescein actin staining (FAS) assays revealed that ΔOI-119 formed fewer pedestals and infected significantly fewer HeLa cells than that of the WT strain (Fig. 1C to E). Accordingly, expression of the seven representative LEE genes—*ler* (master regulator of LEE genes, LEE1), *escT* (LEE1), *escC* (LEE2), *escN* (LEE3), *eae* (intimin, LEE5), *tir* (intimin receptor, LEE5), and *espB* (LEE4)—were markedly downregulated in the ΔOI-119 mutant (Fig. 1F). These results suggest that OI-119 is required for EHEC O157 adherence capacity and LEE gene expression.

10.1128/mBio.02470-20.1 FIG S1 Bacterial growth curves. (A) EHEC O157 WT and ΔOI-119 growth in DMEM medium. (B) EHEC O157 WT, Δ*lmiA*, and Δ*lmiA*(p*lmiA*) growth in DMEM medium. (C) EHEC O157 WT growth in DMEM medium (without Mg^2+^) supplemented with different concentrations of MgCl_2_. O157 WT, EHEC O157 wild-type strain; ΔOI-119, OI-119 mutant; Δ*lmiA*, *lmiA* mutant; Δ*lmiA*(p*lmiA*), *lmiA* mutant complemented with *lmiA*. Data are presented as the means ± SD; *n* = 3. Download FIG S1, TIF file, 0.2 MB. Copyright © 2020 Liu et al. 2020 Liu et al. This content is distributed under the terms of the Creative Commons Attribution 4.0 International license.

10.1128/mBio.02470-20.7 TABLE S1 Expression of OI-119 genes and LEE genes 3 h after incubation of HeLa cells with EHEC O157. Download Table S1, DOCX file, 0.03 MB. Copyright © 2020 Liu et al. 2020 Liu et al. This content is distributed under the terms of the Creative Commons Attribution 4.0 International license.

### LmiA is a positive virulence regulator in OI-119.

OI-119 is a 3.12-kb OI that contains five genes (Fig. S2A); *z4267* (named *lmiA*) encodes a putative DNA-binding protein, *z4268* and *z4269* encode a hypothetical protein with unknown function, and *z4270* and *z4271* encode a putative ATP-binding protein of the ABC transport system. Domain structure analysis revealed that the *lmiA* gene product contains a DUF296 domain (pfam03479; Fig. S2B), which is present in many transcriptional regulators that specifically bind AT-rich DNA sequences. Based on structural comparisons with known metal-binding domains, three highly conserved histidine residues at 85, 87, and 101 on the DUF296 domain appear to form a zinc-binding site (Fig. S2B).

10.1128/mBio.02470-20.2 FIG S2 The effect of *z4268*, *z4269*, *z4270*, and *z4271* on the bacterial adherence capacity and LEE gene expression. (A) Graphical representation of OI-119 structure. The island architecture and nomenclature of the open reading frames (ORFs) refer to the EHEC O157:H7 strain EDL933 sequence (GenBank accession no. AE005174). (B) Domain architecture (top) and predicted structure of LmiA (bottom). The DUF296 domain (pfam03479) was predicted using the NCBI conserved domain database. The LmiA structure was predicted by SWISS-MODEL and visualized by CueMol 2.0. (C) Adhesion of EHEC O157 WT, Δ*z4268*, Δ*z4269*, Δ*z4270*, and Δ*z4271* to Caco-2 cells. (D) qRT-PCR of LEE gene expression changes in EHEC O157 WT, Δ*z4268*, Δ*z4269*, Δ*z4270*, and Δ*z4271*. O157 WT, EHEC O157 wild-type strain; Δ*z4268*, *z4268* mutant; Δ*z4269*, *z4269* mutant; Δ*z4270*, *z4270* mutant; Δ*z4271*, *z4271* mutant. Data are presented as the means ± SD; *n* = 3. Download FIG S2, TIF file, 0.7 MB. Copyright © 2020 Liu et al. 2020 Liu et al. This content is distributed under the terms of the Creative Commons Attribution 4.0 International license.

**FIG 2:**
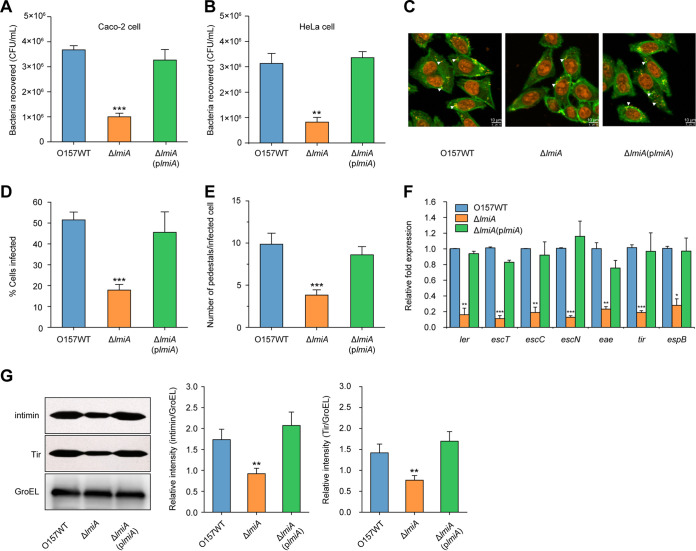
LmiA is a positive virulence regulator of EHEC O157. (A) Adhesion of O157 WT, Δ*lmiA*, and Δ*lmiA*(p*lmiA*) to Caco-2 cells. (B) Adhesion of O157 WT, Δ*lmiA*, and Δ*lmiA*(p*lmiA*) to HeLa cells. (C) FAS assay of HeLa cells infected with O157 WT, Δ*lmiA*, and Δ*lmiA*(p*lmiA*). HeLa nuclei and bacteria were stained with propidium iodide (red), and HeLa cell actin cytoskeleton was stained with FITC-phalloidin (green). Pedestals are observed as green punctate structures associated with bacterial cells and are indicated by arrowheads. Scale bar, 10 μm. (D and E) FAS assay quantification of the percentage of HeLa cells infected (D) and the number of pedestals per infected cell (E). *n *= 300 cells per strain. (F) qRT-PCR of LEE gene expression changes in O157 WT, Δ*lmiA*, and Δ*lmiA*(p*lmiA*). (G) Representative Western blot images and quantitative analysis of intimin and its receptor Tir in O157 WT, Δ*lmiA*, and Δ*lmiA*(p*lmiA*). Protein levels were semiquantified using ImageJ. The relative intensity is shown as the ratio of the signal intensity of intimin or Tir to GroEL (loading control). O157 WT, EHEC O157 wild-type strain; Δ*lmiA*, *lmiA* mutant; Δ*lmiA*(p*lmiA*), *lmiA* mutant complemented with *lmiA*. Data are presented as the means ± SD; *n* = 3. *, *P ≤ *0.05; **, *P ≤ *0.01; ***, *P ≤ *0.001 (Student's *t* test).

Adherence capacity and LEE gene expression levels of ΔOI-119 could be restored to WT levels when an expression plasmid carrying *lmiA* was introduced into ΔOI-119 (Fig. 1), suggesting that *lmiA* is responsible for the virulence defect of this mutant. To further investigate whether *lmiA* is required for the full virulence of EHEC O157, a Δ*lmiA* mutant was generated. The Δ*lmiA* mutant exhibited significantly reduced bacterial adherence, as evidenced by bacterial adherence (Fig. 2A and B) and FAS (Fig. 2C to E) assays, as well as suppressed transcriptional expression of LEE genes compared with that of the WT (Fig. 2F). Growth curves of the EHEC O157 WT, Δ*lmiA* mutant, and complemented strain were similar, eliminating the influence of different growth rates on bacterial adherence and LEE gene expression (Fig. S1B). The Δ*lmiA* mutant defects were comparable to those of ΔOI-119 and were restored to WT levels when complemented with a low-copy-number plasmid carrying *lmiA* (Fig. 2A to F). Furthermore, Western blotting also revealed that the protein levels of intimin (encoded by *eae*) and its receptor Tir were significantly decreased in the Δ*lmiA* mutant compared with those of the WT and Δ*lmiA*-complemented strain (Fig. 2G). These results confirm that LmiA is a positive regulator of EHEC O157 adherence capacity and LEE gene expression. In contrast, deletion of other OI-119 genes, including *z4268*, *z4269*, *z4270*, and *z4271*, had no observable effects on EHEC O157 virulence, as adherence capacity and LEE gene expression levels of these mutants were comparable to those of the WT (Fig. S2C and D).

### LmiA mediates the expression of O157 LEE genes via Ler.

**FIG 3:**
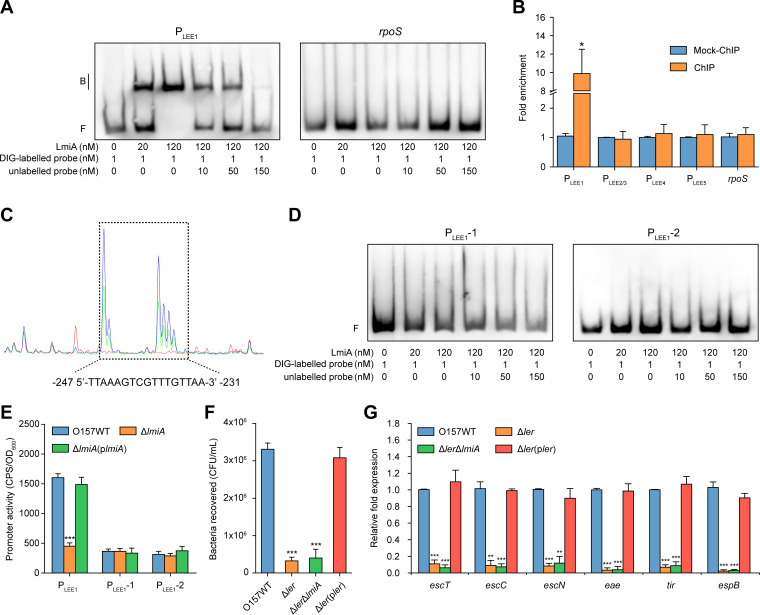
LmiA mediates the expression of EHEC O157 LEE genes via Ler. (A) Gel mobility shift and competition assays of LmiA with the promoter region of LEE1 and *rpoS* (negative control). Positions of the bound (denoted with “B”) and free (denoted with “F”) probes are shown on the left, and the concentrations of the probe and purified LmiA are indicated at the bottom of each lane. (B) Fold enrichment of the promoter region of LEE1, LEE2/3, LEE4, and LEE5 in LmiA-ChIP samples, as measured via ChIP-qPCR. *rpoS* is negative control. (C) LmiA binds to the motif TTAAAGTCGTTTGTTAA in the LEE1 promoter region. FAM-labeled probes (40 nM) were used for binding reactions in the absence (blue peaks) or presence of 2.5 μM (green peaks) or 5.0 μM (red peaks) LmiA. Electropherograms of partially digested DNA from both reactions were merged and analyzed using Peak Scanner software. The region protected from DNase I digestion is boxed, and the corresponding nucleotide sequence is shown beneath the electropherogram. (D) Gel mobility shift and competition assays of LmiA with the modified LEE1 promoter region P_LEE1_-1 (without the binding motif) and P_LEE1_-2 (with the mutated motif, TTAACTGATGGTGTTAA). (E) Promoter activities of P_LEE1_, P_LEE1_-1, and P_LEE1_-2 in O157 WT, Δ*lmiA*, and Δ*lmiA*(p*lmiA*). (F) Adhesion of O157 WT, Δ*ler*, Δ*ler* Δ*lmiA*, and Δ*ler*(p*ler*) to Caco-2 cells. (G) qRT-PCR of LEE gene expression changes in O157 WT, Δ*ler*, Δ*ler* Δ*lmiA*, and Δ*ler*(p*ler*). O157 WT, EHEC O157 wild-type strain; Δ*ler*, *ler* mutant; Δ*ler* Δ*lmiA*, *ler/lmiA* double mutant; Δ*ler*(p*ler*), *ler* mutant complemented with *ler*. Data are presented as the means ± SD; *n* = 3. *, *P ≤ *0.05; **, *P ≤ *0.01; ***, *P ≤ *0.001 (Student's *t* test).

To investigate whether LmiA regulates LEE genes directly or indirectly, we determined LmiA binding to LEE promoters (P_LEE1_, P_LEE2/3_, P_LEE4_, and P_LEE5_) *in vitro* using electrophoretic mobility shift assays (EMSAs) and competition assays. At increasing concentrations of LmiA protein, slow migrating bands were observed for the LEE1 promoter (Fig. 3A). Moreover, addition of the unlabeled LEE1 promoter effectively competed for LmiA binding to the labeled LEE1 promoter (Fig. 3A). These results indicate that LmiA binds specifically to the promoter region of LEE1 *in vitro*. Meanwhile, LmiA did not bind to the negative-control *rpoS* and other LEE promoters under the same experimental conditions (Fig. 3A and Fig. S3A). We next determined the fold enrichment of these LEE promoters in LmiA-chromatin immunoprecipitation (ChIP) samples compared with mock-ChIP control DNA using ChIP-qPCR analysis. In agreement with the EMSA results, P_LEE1_ showed a 9.87-fold enrichment in LmiA-ChIP samples compared with mock-ChIP control samples (Fig. 3B), indicating LmiA binding to P_LEE1_ *in vivo*. In contrast, the enrichment of P_LEE2/3_, P_LEE4_, P_LEE5_, and *rpoS* was similar in both LmiA-ChIP and mock-ChIP samples (Fig. 3B). Using a dye-based DNase I foot-printing assay, we further found that LmiA binds to a specific sequence containing a 17-bp motif (5ʹ-TTAAAGTCGTTTGTTAA-3′), which is located −247 to −231 bp from the proximal transcriptional start site (TSS; Fig. 3C and Fig. S3B). Deletion of the binding motif (P_LEE1_-1) or mutation of the binding motif to TTAACTGATGGTGTTAA (P_LEE1_-2) completely abolished LmiA binding to P_LEE1_ as determined by EMSA (Fig. 3D); these findings indicate that the motif is crucial to the binding ability of LmiA. To further test this *in vivo*, a *lux* reporter assay was used to measure the activity of P_LEE1_, P_LEE1_-1, and P_LEE1_-2 in EHEC O157 WT, the Δ*lmiA* mutant, and the complemented strain. The expression of P_LEE1_-*lux* in the Δ*lmiA* mutant was 4.5-fold lower than in the EHEC O157 WT or its complemented strain (Fig. 3E). In contrast, *lmiA* deletion had no effect on the expression of P_LEE1_-1-*lux* and P_LEE1_-2-*lux*, as lux activity in the Δ*lmiA* mutant was comparable to that of the EHEC O157 WT and its complemented strain (Fig. 3E).

10.1128/mBio.02470-20.3 FIG S3 LmiA did not bind to the promoter regions of LEE2/3, LEE4, and LEE5 both *in vitro* and *in vivo*. (A) Gel mobility shift and competition assays of LmiA with the promotor regions of LEE2/3, LEE4, and LEE5. Positions of the free (denoted with “F”) probes are shown on the left, and the concentrations of the probe and purified LmiA are indicated at the bottom of each lane. (B) DNA sequence of LEE1 (*ler*) promoter region, including TSSs, −10 and −35 recognition sequences, and translational start site. The rectangle indicates the sequence protected by LmiA in the DNase I footprinting assay. (C) qRT-PCR of changes in the expression of *escT* in O157 WT, Δ*ler*, and Δ*ler*Δ*lmiA*. (D) Adhesion of O157 WT, Δ*lmiA*, Δ*lmiA*(p*ler*), Δ*ler* and Δ*ler*(p*lmiA*) to Caco-2 cells. (E) qRT-PCR of LEE gene expression changes in O157 WT, Δ*lmiA*, Δ*lmiA*(p*ler*), Δ*ler*, and Δ*ler*(p*lmiA*). O157 WT, EHEC O157 wild-type strain; Δ*ler*, *ler* mutant; Δ*ler* Δ*lmiA*, *ler/lmiA* double mutant; Δ*lmiA*, *lmiA* mutant; Δ*lmiA*(p*ler*), *lmiA* mutant complemented with *ler*; Δ*ler*(p*lmiA*), *ler* mutant complemented with *lmiA*. Data are presented as the means ± SD; *n* = 3. **, *P ≤ *0.01; ***, *P ≤ *0.001 (Student’s *t*-test). Download FIG S3, TIF file, 1.0 MB. Copyright © 2020 Liu et al. 2020 Liu et al. This content is distributed under the terms of the Creative Commons Attribution 4.0 International license.

The first gene within LEE1 encodes the master regulator Ler, which activates the expression of LEE1 to LEE5 genes. Considering that LmiA can directly bind to the LEE1 promoter and positively regulate *ler* expression (Fig. 3A and Fig. 2F), we investigated whether LmiA regulates EHEC O157 adherence and LEE gene expression via Ler. Inactivation of *ler* in EHEC O157 resulted in a significant reduction of bacterial adherence capacity and LEE gene expression levels (Fig. 3F and G), verifying *ler* as a positive regulator of bacterial virulence. In addition, the ability of the Δ*ler* Δ*lmiA* double mutant to adhere to Caco-2 cells and express LEE genes showed no significant difference from that of the Δ*ler* mutant (Fig. 3F and G and Fig. S3C). In other words, *lmiA* deletion had no effect on EHEC O157 adherence and LEE gene expression in a Δ*ler* background. Complementation of the Δ*lmiA* mutant with *ler* restored bacterial adherence capacity and LEE gene expression to the WT level (Fig. S3D and E), whereas no apparent changes in these virulence features were observed when the Δ*ler* mutant was complemented with *lmiA* (Fig. S3D and E). These results indicate that the regulatory role of LmiA regarding adherence and LEE gene expression in EHEC O157 is mediated by Ler.

### PhoP directly binds to the *lmiA* promoter to activate *lmiA* expression.

**FIG 4:**
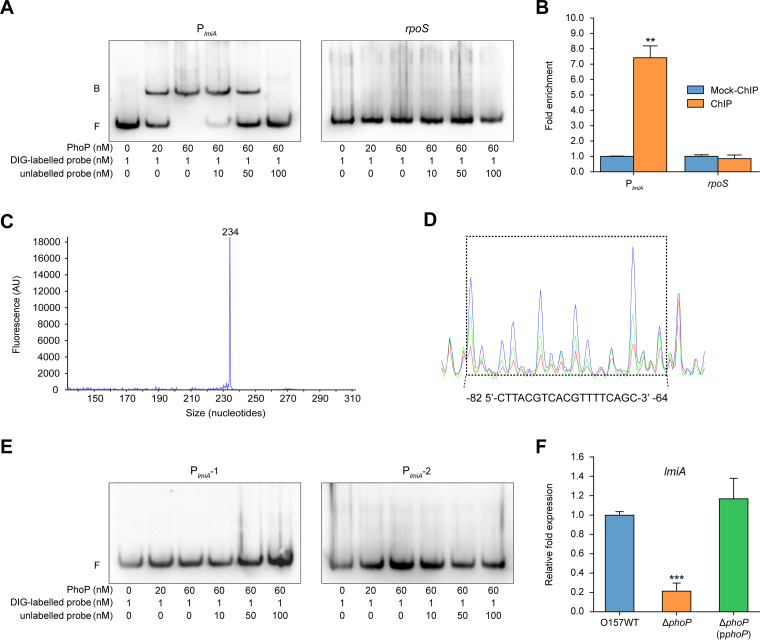
PhoP directly binds to the *lmiA* promoter to activate *lmiA* expression. (A) Gel mobility shift and competition assays of PhoP with the promoter region of *lmiA* and *rpoS* (negative control); positions of the bound (denoted with “B”) and free (denoted with “F”) probes are shown on the left, and the concentrations of the probe and purified PhoP are indicated at the bottom of each lane. (B) Fold enrichment of the promoter region of *lmiA* in PhoP-ChIP samples, as measured via ChIP-qPCR. *rpoS* is negative control. (C) Determination of the TSS of *lmiA* via fluorescent primer extension and comparing retention time of the FAM-labeled primer extension product with that of DNA size standards. (D) PhoP binds to the motif CTTACGTCACGTTTTCAGC in the *lmiA* promoter region. FAM-labeled probes (40 nM) were used for binding reactions in the absence (blue peaks) or presence of 2.5 μM (green peaks) or 5.0 μM (red peaks) PhoP. Region protected from DNase I digestion is boxed and the corresponding nucleotide sequence are shown beneath the electropherogram. (E) Gel mobility shift and competition assays of PhoP with the modified *lmiA* promoter region P*_lmiA_*-1 (without the potential PhoP box) and P*_lmiA_*-2 (with the mutated potential PhoP box, GACCCTCGTCACCAACT). (F) qRT-PCR of changes in the expression of *lmiA* in O157 WT, Δ*phoP*, and Δ*phoP*(p*phoP*). O157 WT, EHEC O157 wild-type strain; Δ*phoP*, *phoP* mutant; Δ*phoP*(p*phoP*), *phoP* mutant complemented with *phoP*. Data are presented as the means ± SD; *n* = 3. **, *P ≤ *0.01; ***, *P ≤ *0.001 (Student's *t* test).

To identify proteins that are involved in the transcriptional regulation of *lmiA*, we performed DNA affinity pulldown assays using a biotin-labeled fragment within the *lmiA* promoter as a probe. This screening led to the discovery of one candidate regulator, PhoP, a two-component transcriptional regulatory protein (Fig. S4A). To further confirm binding ability, EMSA and competition assays were performed with the *lmiA* promoter and PhoP. At increasing concentrations of PhoP protein, slow migrating bands were observed for the *lmiA* promoter (Fig. 4A), whereas no retarded bands were observed for *rpoS* (negative control; Fig. 4A). Addition of the unlabeled *lmiA* promoter led to effective competition with PhoP for binding onto the labeled *lmiA* promoter, and the retarded band disappeared in the presence of 100-fold excess unlabeled DNA (Fig. 4A), indicating that PhoP binds specifically to the *lmiA* promoter *in vitro*. ChIP-qPCR analysis further demonstrated PhoP binding to the *lmiA* promoter *in vivo* (Fig. 4B). Additionally, the *lmiA* promoter was markedly enriched in PhoP-ChIP samples, with a 7.45-fold increase in relative quantity compared with that of the mock-ChIP control samples; the negative-control *rpoS* was not enriched in the PhoP-ChIP samples (Fig. 4B). These results suggest that PhoP binds specifically to the *lmiA* promoter both *in vitro* and *in vivo*.

10.1128/mBio.02470-20.4 FIG S4 (A) SDS-PAGE and MS results of EHEC O157 proteins fished out by DNA affinity pulldown assays using the *lmiA* promoter DNA as bait. Enriched proteins were excised from the SDS-PAGE gel and identified by MALDI-TOF MS/MS. Lane M, molecular mass marker; lane P, proteins eluted with 500 mM NaCl solution. (B) Sequence of the *lmiA* promoter region. The TSS (position +1), putative −10 and −35 regions of the *lmiA* promoter, and the FAM-labeled extension primer sequence are indicated. The potential PhoP box is highlighted in yellow background, and the sequence protected by PhoP in the DNase I footprinting assay is indicated by a rectangle. Download FIG S4, TIF file, 0.6 MB. Copyright © 2020 Liu et al. 2020 Liu et al. This content is distributed under the terms of the Creative Commons Attribution 4.0 International license.

The TSS of the *lmiA* gene was determined by fluorescent primer extension analysis using a fluorescently labeled primer that anneals to the *lmiA* gene. Based on the size of the major product obtained (Fig. 4C), the TSS of the *lmiA* gene was mapped at a T residue located 90 nucleotides upstream from the ATG start codon of *lmiA* (Fig. 4C and Fig. S4B). Putative −35 (TTGCAG) and −10 (TAGCAAAAT) sequences were found upstream of the TSS (Fig. S4B). Using a dye-based DNase I footprinting assay, we further found that PhoP binds to a specific sequence containing a 19-bp motif (5ʹ-CTTACGTCACGTTTTCAGC-3′) located −82 to −64 bp from the TSS of *lmiA* (Fig. 4D and Fig. S4B). PhoP has been shown to bind to the conserved motif (T/G)GTTTANNNNN(T/G)GTTTA, which is termed the PhoP box and is present in the promoter region of many PhoP-activated genes in E. coli (24). Searching the *lmiA* promoter region for this conserved motif led to identification of a potential PhoP box (GACTTACGTCACGTTTT) located −84 to −68 bp from the TSS of *lmiA*, which partially overlapped with the PhoP-binding site determined using the DNase I footprinting assay (Fig. S4B). To further determine whether this potential PhoP box is important for binding to PhoP, we performed EMSAs and competition assays using a P*_lmiA_*-1 DNA fragment (without the potential PhoP box) and a P*_lmiA_*-2 DNA fragment (with the mutated potential PhoP box, GACCCTCGTCACCAACT) under the same conditions. Neither the P*_lmiA_*-1 nor P*_lmiA_*-2 DNA fragment was able to bind to PhoP (Fig. 4E), confirming that the GACTTACGTCACGTTTT motif is crucial to the binding ability of PhoP. Moreover, deletion of *phoP* resulted in significantly decreased expression of *lmiA* (Fig. 4F), indicating that PhoP is a positive transcriptional regulator of *lmiA*. Collectively, all these results demonstrate that PhoP directly and specifically binds to the PhoP box within the *lmiA* promoter region to activate its transcriptional expression.

### Activation of LEE genes by the PhoQ/PhoP two-component regulatory system is mediated by LmiA.

**FIG 5:**
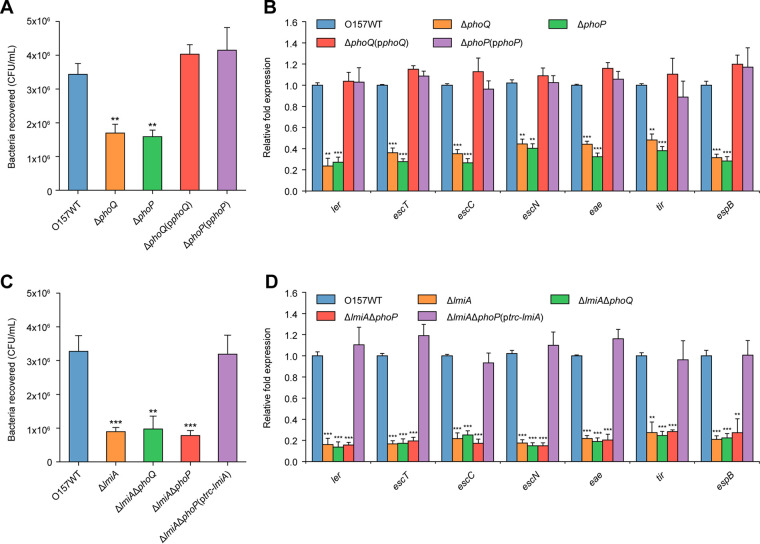
Activation of LEE genes by the PhoQ/PhoP two-component regulatory system is mediated by LmiA. (A) Adhesion of O157 WT, Δ*phoQ*, Δ*phoP*, Δ*phoQ*(p*phoQ*), and Δ*phoP*(p*phoP*) to Caco-2 cells. (B) qRT-PCR of LEE gene expression changes in O157 WT, Δ*phoQ*, Δ*phoP*, Δ*phoQ*(p*phoQ*), and Δ*phoP*(p*phoP*). (C) Adhesion of O157 WT, Δ*lmiA*, Δ*lmiA* Δ*phoQ*, Δ*lmiA* Δ*phoP*, Δ*lmiA* Δ*phoP*(p*trc*-*lmiA*), and Δ*lmiA* Δ*phoP*(p*phoP*) to Caco-2 cells. (D) qRT-PCR of LEE expression in O157 WT, Δ*lmiA*, Δ*lmiA* Δ*phoQ*, Δ*lmiA* Δ*phoP*, Δ*lmiA* Δ*phoP*(p*trc*-*lmiA*), and Δ*lmi* Δ*phoP*(p*phoP*). O157 WT, EHEC O157 wild-type strain; Δ*phoQ*, *phoQ* mutant; Δ*phoP*, *phoP* mutant; Δ*phoQ*(p*phoQ*), *phoQ* mutant complemented with *phoQ*; Δ*phoP*(p*phoP*), *phoP* mutant complemented with *phoP*; Δ*lmiA*, *lmiA* mutant; Δ*lmiA* Δ*phoQ*, *lmiA/phoQ* double mutant; Δ*lmiA* Δ*phoP*, *lmiA/phoP* double mutant; Δ*lmiA* Δ*phoP*(p*trc-lmiA*), *lmiA/phoP* double mutant complemented with *trc* promoter-controlled *lmiA*; Δ*lmiA* Δ*phoP*(p*phoP*), *lmiA/phoP* double mutant complemented with *phoP*. Data are presented as the means ± SD; *n* = 3. *, *P ≤ *0.05; **, *P ≤ *0.01; ***, *P ≤ *0.001 (Student's *t* test).

PhoP and PhoQ constitute a two-component regulatory system where PhoQ is a sensor histidine kinase and PhoP is its cognate DNA-binding transcriptional regulator. Mutation of *phoQ* or *phoP* significantly reduced EHEC O157 bacterial adherence and transcript levels of LEE genes (Fig. 5A and B). Complementation of Δ*phoQ* and Δ*phoP* mutants with the corresponding functional genes restored the defects of both mutant strains to WT levels (Fig. 5A and B). These results suggest that the PhoQ/PhoP two-component system positively regulates EHEC O157 adherence capacity and LEE gene expression.

To further investigate whether the PhoQ/PhoP two-component system regulates EHEC O157 virulence through LmiA, the Δ*phoQ* Δ*lmiA* and Δ*phoP* Δ*lmiA* double-mutant strains were constructed. Adherence capacity and LEE gene expression levels were significantly reduced in all mutants compared with the WT strain (Fig. 5C and D). However, the Δ*lmiA*, Δ*phoQ* Δ*lmiA*, and Δ*phoP* Δ*lmiA* mutants showed similar levels of reduced adherence and LEE gene expression (Fig. 5C and D). Complementation of the Δ*phoP* mutant or Δ*phoP* Δ*lmiA* double mutant with *trc* promoter-controlled *lmiA* fully restored virulence to that of WT levels in the presence of 0.1 mM isopropyl-β-d-thiogalactopyranoside (IPTG) (Fig. 5C and D; Fig. S5A and B). Thus, these results indicate that the PhoQ/PhoP two-component system positively regulates LEE gene expression to promote EHEC O157 adherence and that this regulatory process is mediated by LmiA.

10.1128/mBio.02470-20.5 FIG S5 (A) Adhesion of O157 WT, Δ*phoP*, and Δ*phoP*(p*trc-lmiA*) to Caco-2 cells. (B) qRT-PCR of LEE expression in O157 WT, Δ*phoP*, and Δ*phoP*(p*trc-lmiA*). (C) qRT-PCR of changes in the expression of *lmiA* in O157 WT, Δ*mgrA*, and Δ*grlA*. O157 WT, EHEC O157 wild-type strain; Δ*phoP*, *phoP* mutant; Δ*phoP*(p*trc-lmiA*), *phoP* mutant complemented with *trc* promoter-controlled *lmiA*; Δ*mgrA*, *mgrA* mutant; Δ*grlA*, *grlA* mutant. Data are presented as the means ± SD; *n* = 3. **, *P ≤ *0.01; ***, *P ≤ *0.001 (Student’s *t*-test). Download FIG S5, TIF file, 0.2 MB. Copyright © 2020 Liu et al. 2020 Liu et al. This content is distributed under the terms of the Creative Commons Attribution 4.0 International license.

### Low-magnesium conditions activate LEE gene expression through LimA and the PhoQ/PhoP two-component system.

**FIG 6:**
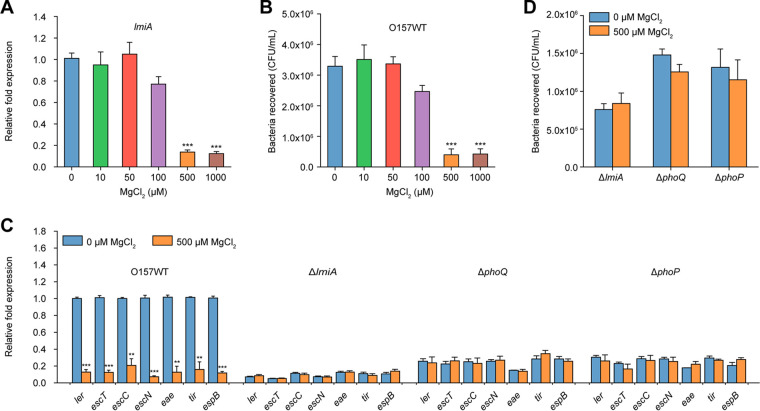
Low magnesium levels activate LEE gene expression to promote EHEC O157 adherence. (A) qRT-PCR of *lmiA* expression levels in O157 WT grown in DMEM (without Mg^2+^) supplemented with different concentrations of MgCl_2_. (B) Adhesion of O157 WT to Caco-2 cells in DMEM (without Mg^2+^) supplemented with different concentrations of MgCl_2_. (C) qRT-PCR of LEE gene expression in O157 WT, Δ*lmiA*, Δ*phoQ*, and Δ*phoP* grown in DMEM (without Mg^2+^) supplemented with 0 or 500 μM MgCl_2_. LEE gene expression in the O157 WT grown in DMEM supplemented with 0 μM MgCl_2_ is represented as 1; all other expression levels are expressed relative to this value. (D) Adhesion of Δ*lmiA*, Δ*phoQ*, and Δ*phoP* to Caco-2 cells in DMEM (without Mg^2+^) supplemented with 0 or 500 μM MgCl_2_. Data are presented as the means ± SD; *n* = 3. **, *P ≤ *0.01; ***, *P ≤ *0.001 (Student's *t* test).

Considering that the PhoQ/PhoP two-component system responds to low magnesium levels as its primary signal for modulating gene expression, we determined the effect of magnesium on *lmiA* expression. *lmiA* transcript levels were not affected under low magnesium levels (<50 μM) but were significantly decreased in the presence of high MgCl_2_ levels (>500 μM; Fig. 6A). Accordingly, bacterial adherence assays revealed that magnesium levels <50 μM had no effect on EHEC O157 adherence, whereas magnesium levels >500 μM significantly decreased EHEC O157 adherence to Caco-2 cells (Fig. 6B). EHEC O157 growth was not affected by magnesium levels, indicating that the negative effect of magnesium on adherence was not due to different growth rates (Fig. S1C). As expected, LEE gene expression levels were also significantly reduced in the presence of high magnesium levels (500 μM) compared with the expression levels observed at 0 μM magnesium (Fig. 6C). These results indicate that EHEC O157 senses low magnesium levels as an important signal for activating LEE gene expression to promote adherence. Furthermore, neither the adherence capacity nor LEE gene expression levels of the Δ*lmiA*, Δ*phoQ*, and Δ*phoP* mutants were affected by high magnesium levels (Fig. 6C and D), indicating that LmiA and the PhoQ/PhoP two-component system are required for this magnesium-induced, virulence-regulatory pathway.

### Low-magnesium conditions promote EHEC O157 colonization in the mouse intestinal tract.

**FIG 7:**
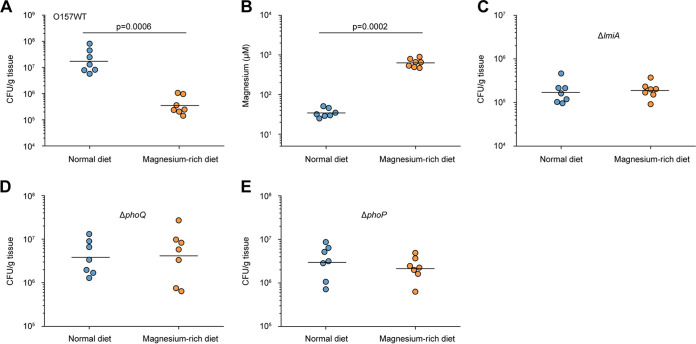
Low magnesium levels promote EHEC O157 colonization in the mouse intestinal tract. (A) Adherence capacity of EHEC O157 WT in the colon of mice fed a normal diet or a magnesium-rich diet. (B) Quantification of magnesium concentrations in the colonic contents obtained from mice fed a normal diet or a magnesium-rich diet. (C to E) Adherence capacity of Δ*lmiA* (C), Δ*phoQ* (D), and Δ*phoP* (E) in the colon of mice fed a normal diet or a magnesium-rich diet. Each graph represents a typical experiment, with 7 mice per group; the horizontal lines represent the geometric means. Statistical significance was assessed via the Mann-Whitney rank-sum test.

The effect of the magnesium status of the host intestinal tract on EHEC O157 adherence was investigated using BALB/c mice fed a normal diet (standard mouse feed and sterilized water) or a magnesium-rich diet (standard mouse feed and sterilized water containing 10 mM MgCl_2_) for 7 days. Under the magnesium-rich diet, the number of bacteria that adhered to the colon was 48.94-fold lower than that obtained from the same sites when fed a normal diet (Fig. 7A). Mice fed a magnesium-rich diet showed an 18.27-fold increase in magnesium levels in the colon luminal content, whereas the magnesium concentration in the colonic content was 500 to 900 μM, which is higher than the concentration required (500 μM) for LEE gene repression (Fig. 7B). Meanwhile, the magnesium concentration in the colonic content under a normal diet was found to be approximately 25 to 50 μM (Fig. 7B). These results indicate that low-magnesium conditions promote EHEC O157 adherence in the mouse intestinal tract and that feeding mice a high-magnesium diet negatively affects adherence *in vivo*. In addition, we found that the number of bacteria recovered from mice infected with Δ*lmiA*, Δ*phoQ*, or Δ*phoP* mutants was much lower than that recovered from EHEC O157 WT-infected mice (Fig. 7C to E). Furthermore, feeding mice a magnesium-rich diet had no clear effects on adherence of these mutants in the mouse intestinal tract (Fig. 7C to E). Therefore, the agreement between our *in vivo* and *in vitro* results further confirms that the effect of magnesium on EHEC O157 adherence is mediated by LmiA and the PhoQ/PhoP two-component system.

### The LmiA-mediated magnesium signaling regulatory pathway is conserved among EHEC and EPEC.

**FIG 8:**
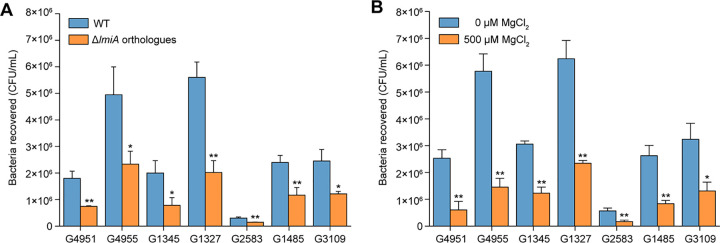
The effect of LmiA and magnesium on the adherence capacity of other EHEC and EPEC strains. (A) Adhesion of different EHEC and EPEC strains, as well as their *lmiA* orthologous gene deletion mutants, to Caco-2 cells. (B) Adhesion of different EHEC and EPEC strains to Caco-2 cells in DMEM (without Mg^2+^) supplemented with 0 or 500 μM MgCl_2_. Data are presented as the means ± SD; *n* = 3. *, *P ≤ *0.05; **, *P ≤ *0.01 (Student's *t* test).

DNA sequence analysis showed that *lmiA* orthologous genes in different lineages of EHEC O157:H7 share 100% nucleic acid sequence identity with *lmiA* (Table S2). The orthologues of *lmiA* were also highly conserved among other sequenced E. coli strains with different pathotypes, including non-O157 EHEC, EPEC, Shiga toxin-producing E. coli, enteroaggregative E. coli, enteroinvasive E. coli, uropathogenic E. coli, neonatal meningitis-associated E. coli, and avian pathogenic E. coli (Table S2). To further investigate whether *lmiA* mediates similar transcriptional regulatory mechanisms in different A/E pathogens, seven representative EHEC and EPEC strains with different serotypes were chosen, after which the corresponding orthologous *lmiA* deletion mutants were constructed (Table S3). Bacterial adherence assays revealed that deletion of *lmiA* orthologous genes also reduced the ability of these EHEC and EPEC strains to adhere to Caco-2 cells (Fig. 8A). Furthermore, adherence of all examined EHEC and EPEC strains to Caco-2 cells was significantly repressed in response to high magnesium levels (500 μM; Fig. 8B). These results suggest that the LmiA-mediated magnesium signaling regulatory pathway is a widespread and highly conserved pathway that is utilized in a range of EHEC and EPEC strains for mediating bacterial virulence.

10.1128/mBio.02470-20.8 TABLE S2 The orthologous genes of *lmiA* in different sequenced Escherichia coli strains. Download Table S2, DOCX file, 0.03 MB. Copyright © 2020 Liu et al. 2020 Liu et al. This content is distributed under the terms of the Creative Commons Attribution 4.0 International license.

10.1128/mBio.02470-20.9 TABLE S3 Strains and plasmids used in this study. Download Table S3, DOCX file, 0.02 MB. Copyright © 2020 Liu et al. 2020 Liu et al. This content is distributed under the terms of the Creative Commons Attribution 4.0 International license.

## DISCUSSION

**FIG 9:**
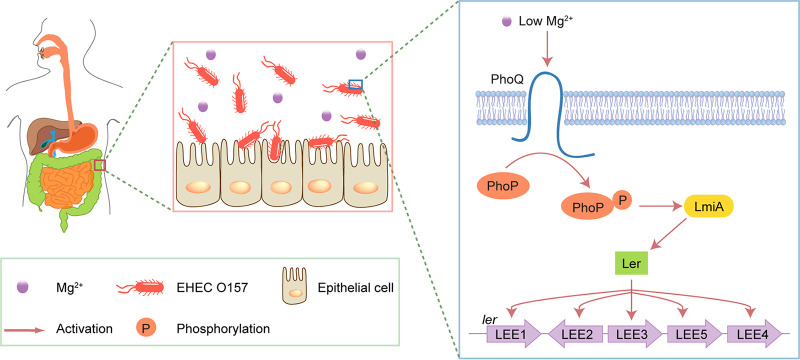
Model for the LmiA-mediated magnesium signaling regulatory pathway in EHEC O157. Under the low-magnesium conditions of the large intestine, PhoQ responds to the low magnesium signal by undergoing autophosphorylation. The phosphate group is then transferred to PhoP, which can directly activate *lmiA* gene transcription. LmiA then activates LEE genes by directly activating Ler, promoting EHEC O157 adherence.

The mammalian intestinal tract provides a complex and competitive environment for microbiota. Successful colonization by pathogens requires scavenging nutrients, competing with commensal bacteria, sensing environmental signals, and precisely regulating virulence gene expression. In this study, we identified a low magnesium-induced signal transduction pathway for the activation of virulence genes that facilitate EHEC O157 colonization in the human large intestine. We proposed a model for this magnesium-dependent regulatory pathway, which is illustrated in Fig. 9; briefly, when EHEC O157 enters the human large intestine where magnesium levels are low, PhoQ responds to the low magnesium signal and undergoes autophosphorylation, after which the phosphate group is transferred to its cognate DNA-binding transcriptional regulator PhoP. The phosphorylated PhoP (PhoP-P) then directly activates *lmiA* gene expression by binding the *lmiA* promoter, after which LmiA activates *ler* (encoding the master regulator of LEE genes) and other LEE genes, promoting bacterial adherence and colonization *in vivo*. The ability of EHEC O157 to express LEE genes and adhere to host epithelial cells (both *in vitro* and *in vivo*) was severely reduced when the magnesium-dependent regulatory pathway was blocked by the deletion of *lmiA* or *phoQ*/*phoP*, indicating that this pathway is critical for EHEC O157 to establish successful colonization.

The core genome of pathogenic bacteria is enhanced with novel genetic islands and small clusters of genes often associated with increased virulence (25). These novel genes located within OIs provide bacteria with a higher level of adaptation that can open up new ecological niches and facilitate efficient dissemination to new hosts (25). Although they have been widely researched, the majority of OIs have not been assigned a function. Herein, we demonstrated that OI-119 is involved in EHEC O157 virulence regulation, as deletion of this OI significantly decreased bacterial ability of adhering to host epithelial cells and expressing LEE genes. Thus, our findings provide insights into OI-119 as well as EHEC O157 OI function.

EHEC O157 regulation of LEE is very complex and is governed by at least four different kinds of regulatory influences (6, 26), including (i) environmental factors such as pH, butyrate, ethanolamine, fucose, and biotin; (ii) regulators encoded within LEE, including the master LEE regulator Ler and the GrlR-GrlA regulatory system; (iii) global regulators, including H-NS, IHF, Fis, and QseA; and (iv) horizontal transfer-acquired regulators such as EivF, EtrA, and GrvA. Our study provides an example of the fourth type of regulatory influence, regulation of LEE gene expression by LmiA encoded in OI-119. LmiA was identified as a novel positive LEE regulator that has a profound effect on EHEC O157 adherence both *in vitro* and *in vivo*, as *lmiA* is an essential element that integrates the low magnesium signal into the LEE regulatory network. Therefore, acquisition of *lmiA* through horizontal transfer was crucial for the evolution of EHEC O157 into a successful intestinal pathogen. Additionally, our findings significantly expand our understanding of the EHEC O157 virulence regulatory mechanism and the increased complexity of the regulatory network governing LEE gene expression. Meanwhile, the other OI-119 genes, including *z4268*, *z4269*, *z4270*, and *z4271*, had no observable effects on EHEC O157 adherence and LEE gene expression (Fig. S2C and D); therefore, we believe that these genes may be redundant or involved in other bacterial processes, which require further investigation.

The LEE1 operon has two promoters, a distal promoter (P1) and a proximal promoter (P2) (27). The LmiA-binding motif identified in this study is located −247 to −231 bp from the proximal TSS (−207 to −191 bp from the proximal −35 region), and −115 to −99 bp from the distal TSS (−78 to −62 bp from the distal −35 region) (Fig. S3B). Although there is a distance of about 60 to 80 bp between the LmiA-binding motif and the −35 region of the distal promoter, these two regions may be topologically close together in the genome. We theorized that LmiA specifically binds to a 17-bp motif to promote LEE1 transcription from the distal promoter by facilitating recruitment of RNA polymerase and/or other transcriptional regulators.

Many PhoP-regulated genes in E. coli share a conserved motif termed the PhoP box at their −35 region with a maximum mismatch of two nucleotides (24). In this study, we detected a potential PhoP box with a mismatch of three nucleotides that partially overlapped with the PhoP-binding site and was located 30 bp upstream from the predicted −35 region of the *lmiA* promoter (Fig. S4B). This potential PhoP box was important for binding to PhoP, as deletion or mutation of this box completely abolished PhoP binding to the *lmiA* promoter (Fig. 4E). In agreement, a previous study also found that PhoP box present in the promoter region of *crcA* exhibits a three-base difference from the consensus PhoP box sequence (28). Furthermore, both the *ugd* and *mgtC* promoters harbor a PhoP box that is located much farther upstream from the −35 region (29). Overall, these results indicate that PhoP can regulate transcription directly from promoters harboring PhoP boxes with a maximum mismatch of three nucleotides and at various distances from the −35 RNA polymerase-binding site, expanding our understanding of the regulatory mechanism of PhoP.

Previous studies revealed that the PhoP/PhoQ two-component regulatory system activates MgrR, which, in turn, stimulates GrlA to enhance LEE1 expression (30, 31). Here, we found that activation of *lmiA* expression by the PhoP/PhoQ two-component regulatory system is independent of MgrR and GrlA, as deletion of *mgrA* or *grlA* had no observable effects on *lmiA* expression (Fig. S5C).

Besides low magnesium levels, mild acidic pH and cationic antimicrobial peptides are also direct activating signals for the PhoQ/PhoP two-component system (32, 33). In the present study, we found that the ability of EHEC O157 to adhere to Caco-2 cells and express *lmiA* and LEE genes was also significantly increased at mild acidic pH (pH <6.0) and in the presence of cationic antimicrobial peptide polymyxin B (>0.5 μg/ml; Fig. S6). However, the human large intestine is a neutral or weakly alkaline environment with a pH range of 7.0 to 7.5 (34). In addition, cationic antimicrobial peptides are mainly produced and secreted by Paneth cells within the crypts of the small intestine, and only a limited number of antimicrobial peptides exist in the large intestine (35, 36). Therefore, mild acidic pH and cationic antimicrobial peptides are unlikely sensed as intestinal signals by EHEC O157 to activate virulence gene expression for promoting bacterial adherence in the large intestine. It is more likely that the PhoQ/PhoP system responds to low magnesium levels as the only signal in the human large intestine to positively regulate EHEC O157 adherence and LEE gene expression.

10.1128/mBio.02470-20.6 FIG S6 The effect of mild acidic pH and polymyxin B on the EHEC O157 adherence capacity and the expression of *lmiA* and LEE genes. (A) qRT-PCR of *lmiA* expression in EHEC O157 WT grown in DMEM at different pH values. (B) Adhesion of EHEC O157 WT to Caco-2 cells in DMEM at different pH values. (C) qRT-PCR of LEE gene expression in EHEC O157 WT grown in DMEM at pH 5.5 or 7.5. (D) qRT-PCR of *lmiA* expression in EHEC O157 WT grown in DMEM supplemented with different concentrations of polymyxin B. (E) Adhesion of EHEC O157 WT to Caco-2 cells in DMEM supplemented with different concentrations of polymyxin B. (F) qRT-PCR of LEE gene expression in EHEC O157 WT grown in DMEM supplemented with 0 or 1.0 μg/ml polymyxin B. Data are presented as the means ± SD; *n* = 3. *, *P ≤ *0.05; **, *P ≤ *0.01; ***, *P ≤ *0.001 (Student’s *t*-test). Download FIG S6, TIF file, 0.4 MB. Copyright © 2020 Liu et al. 2020 Liu et al. This content is distributed under the terms of the Creative Commons Attribution 4.0 International license.

Bioinformatics analysis revealed that *lmiA* is conserved and widespread among different fully sequenced E. coli strains. In addition, most E. coli strains with *lmiA* are important human or animal pathogens, indicating that *lmiA* may play crucial roles during the evolution of nonpathogenic E. coli into pathogenic strains. *lmiA* orthologues in seven representative EHEC and EPEC strains were also found to promote bacterial adherence to host cells, implying that LimA-mediated transcriptional regulation of bacterial virulence is not specific to EHEC O157:H7 but is a general mechanism in many strains. Clearly, besides acquisition of virulence factors, acquisition of novel virulence regulatory mechanisms during evolution also promotes the emergence of important human pathogens.

Diarrheic diseases caused by EHEC and EPEC are a public health problem worldwide, particularly in developing countries. Outbreak surveillance data from the CDC reported that EHEC O157 alone results in more than 73,000 illnesses, 2,200 hospitalizations, and 60 deaths annually in the United States (37). Several therapeutic strategies have been developed, including the use of antibiotics and vaccinations; however, there is no effective treatment for EHEC/EPEC infection, and the use of antibiotics may be contraindicated, as bacterial cell lysis by antibiotics leads to increased toxin release. Therefore, highly effective measures for prevention and control of EHEC/EPEC infections are essential. Our study revealed that feeding mice a magnesium-rich diet significantly reduced EHEC O157 adherence in the mouse intestine. This supports the possibility of using magnesium as a dietary supplement or antivirulence drug for the prevention and control of EHEC/EPEC infections; moreover, it is a safer strategy as it does not require bacterial cell lysis. Considering that magnesium may be absolutely absorbed before reaching the human large intestine, slow-release magnesium agents would be preferable for such applications.

In conclusion, our study further enhanced our understanding of how EHEC utilizes environmental cues to facilitate intestinal colonization and offers a paradigm of environmental signal sensing and virulence regulation that can be exploited for researching other human gastrointestinal bacterial pathogens. Our findings also support the potential of magnesium supplements as a strategy against EHEC/EPEC infections.

## MATERIALS AND METHODS

### Ethics statement.

All animal experiments were performed in accordance with the standards established in the Guide for the Care and Use of Laboratory Animals published by the Institute of Laboratory Animal Resources (ILAR) of the National Research Council (United States). Animal experimental protocols were approved by the Institutional Animal Care Committee of Nankai University and Tianjin Institute of Pharmaceutical Research New Drug Evaluation Co. Ltd. (IACUC no. 2016032102), Tianjin, China. Every effort was made to minimize animal suffering and reduce the number of animals used.

### Bacterial strains, plasmids, and growth conditions.

Bacterial strains and plasmids used in this study are summarized in Table S3. Mutant strains were generated by the λ Red recombinase system (38). Target genes were replaced by a kanamycin resistance cassette or chloramphenicol acetyltransferase cassette, after which antibiotic resistance cassettes were subsequently eliminated using pCP20 plasmid. All mutant strains were verified by PCR amplification and sequencing. Complementary strains were constructed by cloning target genes and their own promoter regions into pACYC184, after which the resulting constructs were electroporated into the corresponding mutant strains. *lmiA* was also cloned into plasmid pTrc99A under the control of the IPTG-inducible *trc* promoter and then electroporated into the mutants Δ*phoP* and Δ*phoP* Δ*lmiA*. For LmiA and PhoP purification, *lmiA* and *phoP* were inserted into the pET28a expression vector, after which the resulting constructs were electroporated into E. coli BL21. Primers used for construction of mutant strains and recombinant plasmids are listed in Table S4. All strains were maintained at −80°C in LB broth with 20% glycerol and grown overnight at 37°C in LB broth for experiments. As required, antibiotics were added at the following final concentrations: ampicillin, 100 μg ml^−1^; chloramphenicol, 15 μg ml^−1^; and kanamycin, 50 μg ml^−1^.

10.1128/mBio.02470-20.10 TABLE S4 Primers used in this study (5′ to 3′). Download Table S4, DOCX file, 0.03 MB. Copyright © 2020 Liu et al. 2020 Liu et al. This content is distributed under the terms of the Creative Commons Attribution 4.0 International license.

### Bacterial adherence assays.

Adherence assays were performed using a previously described method (39). Caco-2 and HeLa cells were obtained from the Shanghai Institute of Biochemistry and Cell Biology, Chinese Academy of Sciences (Shanghai, China). Cell cultures were grown at 37°C and 5% CO_2_ until confluent. Prior to infection, Caco-2 or HeLa cells were washed three times with prewarmed phosphate-buffered saline (PBS), and the medium was replaced with fresh Dulbecco modified Eagle medium (DMEM) without antibiotics and fetal bovine serum (FBS). Cell monolayers were then infected with exponential-phase bacterial culture (10^8^ bacteria/well) for 3 h at 37°C in a 5% CO_2_ atmosphere. Nonadherent bacteria were removed by extensive washing (six times) with prewarmed PBS. To collect adherent bacteria, Caco-2 or HeLa cells were disrupted with 0.1% SDS at 37°C for 5 min. The lysate was diluted and then plated on LB agar. The attachment efficiency was determined by counting the CFU per milliliter.

### FAS assays.

HeLa cells were grown on coverslips in 6-well culture plates filled with DMEM supplemented with 10% FBS at 37°C and 5% CO_2_ until reaching 80% confluence. Before infection, the wells were washed with prewarmed PBS and replaced with fresh DMEM without antibiotics and FBS. Overnight bacterial cultures were diluted 1:100 to infect HeLa cells. After a 6-h infection at 37°C and 5% CO_2_, the coverslips were washed three times with prewarmed PBS, fixed with 4% paraformaldehyde, permeabilized with 0.01% Triton X-100, and treated with fluorescein isothiocyanate (FITC)-labeled phalloidin to visualize actin accumulation and propidium iodide to visualize host cell nuclei and bacteria. The coverslips were mounted on slides and imaged with a Zeiss LSM 800 microscope (Zeiss, Oberkochen, Germany).

### qRT-PCR.

Total RNA was isolated from samples using the TRIzol LS reagent (catalog no. 10296028; Invitrogen, Carlsbad, CA) and treated with RNase-free DNase I to eliminate genomic DNA contamination. cDNA was synthesized using the PrimeScript 1st strand cDNA synthesis kit (catalog no. D6110A; TaKaRa Bio, Kusatsu, Japan) according to the manufacturer’s instructions. qRT-PCR analysis was conducted using SYBR green PCR master mix (catalog no. 4367659; Applied Biosystems, Foster City, CA) on an ABI 7300 real-time PCR system (Applied Biosystems). To normalize differences in total RNA quantity among samples, the 16S rRNA gene was used as a reference control. The relative difference in gene expression was calculated using the threshold cycle (ΔΔ*CT*) method (40).

### Western blot analysis.

Bacterial cultures were grown at 37°C in LB broth to an optical density at 600 nm (OD_600_) ≈ 1.0. Whole cells were harvested and washed three times with PBS (pH 7.4), after which the bacterial cells were resuspended in ∼100 μl (normalized for OD_600_) SDS-PAGE solubilization buffer (62.5 mM Tris-HCl [pH 6.8], 10% [vol/vol] glycerol, 5% [wt/vol] SDS, 5% [vol/vol] 2-mercaptoethanol, and 0.002% [wt/vol] bromophenol blue) and lysed at 100°C for 10 min. Proteins were separated via 12% SDS-PAGE before transfer to polyvinylidene difluoride (PVDF) membranes. The PVDF membranes were blocked with Tris-buffered saline with Tween 20 (TBST) containing 5% nonfat milk for 1 h at room temperature. The proteins were detected using the primary antibodies anti-FLAG (1:2,500; catalog no. F1804; Sigma-Aldrich, St. Louis, MO) or anti-GroEL (1:1,000; catalog no. ab82592; Abcam, Cambridge, UK), and the secondary antibody goat anti-mouse horseradish peroxidase (HRP) (1:5,000; catalog no. CW0102; CWBio, Beijing, China), followed by visualization with an ECL (enhanced chemiluminescence) reagent (catalog no. CW0048M; CWBio, Beijing, China). Protein levels were semiquantified via densitometry using ImageJ software (National Institutes of Health, Bethesda, MD). Relative intensity was indicated as the ratio of the protein signal intensity of intimin or Tir to GroEL (loading control). Three independent experiments were performed.

### Mouse colonization experiments.

All animal experiments were performed according to the standards set forth in the *Guide for the Care and Use of Laboratory Animals* (41). The experimental protocols were approved by our institutional animal care committee. Six-week-old female BALB/c were purchased from Vital River Laboratory Animal Technology Co. Ltd. (Beijing, China). Mice were fed either a normal diet (standard mouse feed and sterilized water) or magnesium-rich diet (standard mouse feed and sterilized water containing 100 mM MgCl_2_) *ad libitum* for 7 days. For each group, 7 mice were orally infected by pipette feeding of 100 μl PBS containing 10^9^ CFU of bacteria in the logarithmic phase of growth. Infected mice were anesthetized and euthanized by cervical dislocation 6 h after infection. The ileum and colon were then excised from the infected animals, and each part of the intestine was washed three times vigorously with PBS. The intestinal tissues were weighed and homogenized in 0.5 ml PBS, after which the homogenates were diluted and plated on LB agar containing nalidixic acid (50 μg ml^−1^). The *in vivo* attachment efficiency was determined by counting the number of CFU per gram of organs.

### Gel mobility shift assays.

We purified 6×His N-terminal-tagged LmiA and 6×His N-terminal-tagged PhoP using nickel columns (item no. 17057501; GE Healthcare, Chicago, IL). DNA fragments containing the LEE1, LEE2 to LEE3, LEE4, and LEE5 promoter regions were amplified by PCR using EHEC O157:H7 strain EDL933 genomic DNA as a template and the primers described by Branchu et al. (42) and Kendall et al. (43). Gel shift assays and competition assays were performed using the DIG Gel Shift kit, second generation (catalog no. 03353591910; Roche, Basel, Switzerland). In each case, 1 nM of each DNA probe was incubated with increasing concentrations of proteins in binding buffer (1 mM Tris-HCl [pH 7.5], 0.1 mM EDTA, 0.2 mM dithiothreitol [DTT], 5 mM MgCl_2_, and 10 mM KCl). For PhoP gel mobility shift assays, 0.1 M acetyl phosphate was added to the binding buffer for generating phosphorylated, active PhoP (44). For competition assays, various concentrations of unlabeled DNA fragments (10 to 100 nM) were added. The reaction mixtures were incubated for 20 min at 37°C. Samples were separated by 6% or 8% nondenaturing polyacrylamide gel and transferred to nylon membranes. Labeled fragments were visualized using Amersham Imager 600 (GE Healthcare).

### ChIP-qPCR.

ChIP was performed as previously described (45, 46) with some modifications. Bacterial cultures were grown to the exponential phase (OD_600_ ≈ 0.6) in LB broth. Formaldehyde was added to a final concentration of 1% and incubated at room temperature for 25 min. To quench the cross-linking reaction, glycine was added to a final concentration of 0.5 M. Cross-linked cells were harvested by centrifugation and washed three times with ice-cold TBS. Washed cells were resuspended in 0.5 ml lysis buffer (10 mM Tris, pH 8.0, 100 mM NaCl, 1 mM EDTA, 0.5 mM EGTA, 0.1% deoxycholate, 0.5% *n*-lauroylsarcosine, and 1 mg/ml lysozyme) and incubated at 37°C for 30 min. Following lysis, 0.5 ml IP buffer (50 mM HEPES-KOH, pH 7.5, 150 mM NaCl, 1 mM EDTA, 1% Triton X-100, 0.1% sodium deoxycholate, 0.1% SDS, and 1 mM phenylmethylsulfonyl fluoride [PMSF]) was added, and the cells were sonicated once for 30 s with a needle sonicator, after which unlysed debris was pelleted by centrifugation. DNA was sheared to an average size of ∼500 bp using a sonicator for 20 min with a 10-s on/10-s off cycle. Cell debris was pelleted by centrifugation at 16,000 × *g* for 10 min at 4°C, after which the resulting supernatant was used for IP with anti-3×FLAG antibody (catalog no. F1804; Sigma-Aldrich) and protein A magnetic beads (catalog no. 10002D; Invitrogen). As a negative control, ChIP was performed using an aliquot without the addition of any antibodies. After 5 h of incubation at 4°C, the beads were washed 5 times with RIPA buffer (50 mM HEPES pH 7.5, 500 mM LiCl, 1 mM EDTA, 1% Nonidet P-40, and 0.7% deoxycholate), washed once in Tris-EDTA (pH 8.0) plus 50 mM NaCl, and finally eluted in 100 μl elution buffer (50 mM Tris-HCl, pH 7.5, 10 mM EDTA, and 1% SDS). The eluted samples were incubated overnight at 65°C for reverse cross-linking, followed by incubation with RNaseA for 2 h at 37°C and proteinase K solution (catalog no. AM2546; Invitrogen) for 2 h at 55°C to degrade RNA and protein, respectively. The DNA samples were then purified with a PCR purification kit (catalog no. 28104; Qiagen, Hilden, Germany). To measure the enrichment of P_LEE1_, P_LEE2/3_, P_LEE4_, and P_LEE5_ in the immunoprecipitated DNA samples, relative-abundance qPCR was performed with Fast SYBR green master mix; 16S rRNA gene was used as a reference control. Relative enrichment was calculated as fold change using the ΔΔ*CT* method (40).

### Fluorescent primer extension.

A 6-carboxyfluorescein (FAM)-labeled oligonucleotide complementary to the sequence 120 to 141 bp downstream, the initiation codon of the *lmiA* gene was used for primer extension reactions. Total RNA (2 μg) and 1 μM FAM-labeled primer were incubated at 70°C for 5 min, followed by reverse transcription at 42°C for 60 min. Samples were then incubated with 20 μg RNase A at 37°C for 30 min, after which cDNA was ethanol precipitated and dissolved in 15 μl nuclease-free water. Finally, the products were analyzed using the ABI 3730 genetic analyzer.

### Dye primer-based DNase I footprinting assay.

The promoter region of LEE1 and *lmiA* was amplified using EHEC O157:H7 EDL933 as a template and the 6-FAM-labeled forward primers (with 6-FAM modification at the 5ʹ end) and reverse primers. The 6-FAM-labeled probe (40 nM) was then incubated with 2.5 or 5.0 μM LmiA or PhoP in band-shift buffer, after which the protein-DNA mixture was partially digested with 0.05 units of DNase I for 5 min at 25°C. The reaction was quenched by 0.25 M EDTA and purified using the Qiaquick PCR purification kit (catalog no. 28104; Qiagen). Control samples were prepared without protein, and genotype samples were analyzed using the ABI 3730 genetic analyzer.

### Luminescence screening assays.

For *lux* reporter fusion, P_LEE1_, P_LEE1_-1, and P_LEE1_-2 were cloned into the pMS402 plasmid (XhoI/BamHI). The *lux* reporters were then transformed into EHEC O157 WT, Δ*lmiA* mutant, and its complemented strain. Expression of *lux*-based reporters from cells grown in liquid culture was measured as counts per second (CPS) of light production. The lux activity value was obtained by CPS/OD_600_.

### DNA affinity pulldown assay.

DNA pulldown assays were performed as previously described (47) with minor modifications. Briefly, the biotin-labeled DNA fragment containing the promoter region of *lmiA* was amplified from EHEC O157:H7 strain EDL933 genomic DNA. The biotinylated bait DNA was bound to streptavidin-coated Dynabeads (catalog no. 11205D; Invitrogen), followed by incubation with crude extracts obtained from EHEC O157:H7 WT cells. After washing extensively with the nonspecific competitor poly(dI-dC) at a low salt concentration, bound proteins were released by elution buffer containing 500 mM NaCl. The eluted proteins were separated by SDS-PAGE and stained with Coomassie brilliant blue. Proteins were excised from the gel and analyzed by matrix-assisted laser desorption ionization–time-of-flight tandem mass spectrometry (MALDI-TOF MS/MS) analysis after tryptic digestion. Sequence and peptide fingerprint data were analyzed using the NCBI database.

### Quantification of magnesium levels in mouse intestinal tracts.

Six-week-old female BALB/c mice were fed a normal diet or magnesium-rich diet *ad libitum* for 7 days. In each group, 12 mice were euthanized to obtain the luminal contents. The luminal contents were homogenized in 1 ml distilled water and centrifuged at 12,000 × *g* and 4°C for 10 min. The supernatant was collected and diluted to appropriate concentrations. Magnesium quantification was performed using the magnesium assay kit (catalog no. MAK026; Sigma-Aldrich) according to the manufacturer’s instructions.

### Statistical analysis.

Statistical analysis was conducted using MedCalc software (v12.3.0.0). The data represent the mean ± standard deviation (SD) of three independent experiments. Differences between two mean values were evaluated by a two-tailed Student's *t* test. For mouse colonization experiments, statistical significance was assessed via the Mann-Whitney rank-sum test. *P* values of <0.05 were considered statistically significant.
