# Supplementary material for: Magnesium Sensing Regulates Intestinal Colonization of Enterohemorrhagic Escherichia coli O157:H7
Source: mBio. 2020 Nov 10;11(6):e02470-20. doi: 10.1128/mBio.02470-20 (PMC7667037; doi:10.1128/mBio.02470-20)
Supplement: TABLE S1 [file mBio.02470-20-st001.docx]

**Table S1. Expression of OI-119 genes and LEE genes 3 h after incubation of HeLa cells with EHEC O157**

| Gene | Product | RPKM  DMEM-grown O157 | RPKM  HeLa-attached O157 | Fold^*^ | *P* value | Fold^†^ |
| --- | --- | --- | --- | --- | --- | --- |
| *z4267* | Putative DNA-binding protein | 32.87 | 1.87 | -17.55 | 2.31E-04 | -10.42 ± 1.73 |
| *z4268* | Hypothetical protein | 30.44 | 0.00 | 0.00 | 1.45E-05 | -12.48 ± 3.45 |
| *z4269* | Hypothetical protein | 26.85 | 7.42 | -3.62 | 3.00E-02 | -6.10 ± 1.25 |
| *z4270* | Putative ATP-binding protein of the ABC transport system | 19.79 | 9.22 | -2.15 | 4.70E-02 | -7.52 ± 2.54 |
| *z4271* | Putative ATP-binding protein of the ABC transport system | 19.00 | 6.39 | -2.98 | 3.38E-02 | -9.07 ± 1.33 |
| *z5102* | hypothetical protein | 17.27 | 0.00 | 0.00 | 5.30E-04 |  |
| *escF* | Type III secretion apparatus needle protein | 111.01 | 0.00 | 0.00 | 3.47E-10 |  |
| *cesD2* | hypothetical protein | 318.81 | 57.75 | -5.52 | 1.92E-04 |  |
| *espB* | secreted protein EspB | 209.04 | 96.90 | -2.16 | 5.25E-02 | -4.65 ± 0.63 |
| *espD* | secreted protein EspD | 26.10 | 36.36 | 1.39 | 8.01E-01 |  |
| *espA* | EspA | 35.06 | 0.00 | 0.00 | 4.28E-06 |  |
| *sepL* | hypothetical protein | 47.28 | 23.78 | -1.99 | 1.22E-01 |  |
| *escD* | Pas | 83.51 | 8.40 | -9.94 | 2.28E-05 |  |
| *eae* | Gamma intimin | 81.53 | 15.51 | -5.26 | 1.31E-03 | -6.35 ± 0.74 |
| *tir* | Translocated intimin receptor Tir | 45.05 | 19.21 | -2.35 | 3.42E-02 | -4.13± 0.80 |
| *map* | hypothetical protein | 139.15 | 5.03 | -27.66 | 8.12E-08 |  |
| *cesF* | hypothetical protein | 168.28 | 56.43 | -2.98 | 1.13E-02 |  |
| *sepQ* | SepQ | 74.17 | 12.46 | -5.95 | 5.20E-04 |  |
| *z5118* | hypothetical protein | 66.59 | 0.00 | 0.00 | 2.87E-08 |  |
| *escV* | hypothetical protein | 168.28 | 56.43 | -2.98 | 1.13E-02 |  |
| *z5121* | hypothetical protein | 42.15 | 0.00 | 0.00 | 1.22E-06 |  |
| *sepZ* | SepZ | 597.45 | 336.06 | -1.78 | 6.72E-02 |  |
| *z5123* | hypothetical protein | 47.76 | 26.48 | -1.80 | 1.52E-01 |  |
| *escJ* | EscJ | 42.51 | 0.00 | 0.00 | 1.03E-06 |  |
| *escC* | EscC | 14.71 | 0.00 | 0.00 | 1.01E-03 | -6.22 ± 0.91 |
| *cesD* | Type III secretion low calcium response chaperone LcrH/SycD | 3.98 | 0.00 | ND | ND |  |
| *grlA* | hypothetical protein | 17.61 | 0.00 | 0.00 | 3.91E-04 |  |
| *grlR* | hypothetical protein | 704.86 | 126.97 | -5.55 | 1.36E-04 |  |
| *escU* | secretion system apparatus protein SsaU | 14.72 | 10.39 | -1.42 | 4.26E-01 |  |
| *escT* | escT | 4.89 | 3.48 | ND | ND | -3.69 ± 0.57 |
| *escS* | EscS | 0.00 | 9.23 | ND | ND |  |
| *z5136* | hypothetical protein | 52.87 | 8.80 | -6.01 | 1.10E-03 |  |
| *z5137* | hypothetical protein | 68.95 | 17.63 | -3.91 | 5.24E-03 |  |
| *z5138* | hypothetical protein | 3.03 | 8.65 | ND | ND |  |
| *cesAB* | hypothetical protein | 16.12 | 25.07 | 1.56 | 6.52E-01 |  |
| *ler* | hypothetical protein | 79.77 | 34.56 | -2.31 | 5.45E-02 | -4.46 ± 0.85 |
| *espG* | hypothetical protein | 164.00 | 5.22 | -31.43 | 6.90E-09 |  |

*Fold change measured via comparative transcriptome analysis (Yang B, 2015).

^†^Fold change measured via qRT-PCR; the values represent the mean ± SD of three independent experiments (This study).

-, downregulated.

ND, the RPKM <10 in both compared samples.

*P* values were calculated using the binomial test.
