# Supplementary material for: Magnesium Sensing Regulates Intestinal Colonization of Enterohemorrhagic Escherichia coli O157:H7
Source: mBio. 2020 Nov 10;11(6):e02470-20. doi: 10.1128/mBio.02470-20 (PMC7667037; doi:10.1128/mBio.02470-20)
Supplement: TABLE S2 [file mBio.02470-20-st002.docx]

**Table S2. The orthologous genes of *lmiA* in different sequenced *Escherichia coli* strains**

|  |  |  | Identical with *lmiA* (%) | |
| --- | --- | --- | --- | --- |
| Strains | Pathotypes* | Orthologous gene | Nucleic acid | Amino acid |
| *Escherichia coli* O157:H7 Sakai | EHEC | ECs3799 | 100 | 100 |
| *Escherichia coli* O157:H7 TW14359 | EHEC | ECSP_3894 | 100 | 100 |
| *Escherichia coli* O157:H7 TW14588 | EHEC | - | 100 | 100 |
| *Escherichia coli* O157:H7 Xuzhou21 | EHEC | CDCO157_RS19275 | 100 | 100 |
| *Escherichia coli* O157:H7 EC4115 | EHEC | ECH74115_4225 | 100 | 100 |
| *Escherichia coli* O157:H7 4276 | EHEC | A4C51_04665 | 100 | 100 |
| *Escherichia coli* O157:H7 3384 | EHEC | A4C38_04690 | 100 | 100 |
| *Escherichia coli* O157:H7 9234 | EHEC | A4C47_04665 | 100 | 100 |
| *Escherichia coli* O157:H7 2159 | EHEC | A4C45_04665 | 100 | 100 |
| *Escherichia coli* O157:H7 8368 | EHEC | A4C39_04695 | 100 | 100 |
| *Escherichia coli* O157:H7 2149 | EHEC | A4C44_04665 | 100 | 100 |
| *Escherichia coli* O157:H7 1130 | EHEC | A4C50_04675 | 100 | 100 |
| *Escherichia coli* O157:H7 FRIK944 | EHEC | A9L45_21320 | 100 | 100 |
| *Escherichia coli* O157:H7 FRIK2455 | EHEC | A8V32_20125 | 100 | 100 |
| *Escherichia coli* O157:H7 FRIK2533 | EHEC | A8V31_20415 | 100 | 100 |
| *Escherichia coli* O157:H7 FRIK2069 | EHEC | A8V30_20425 | 100 | 100 |
| *Escherichia coli* 28RC1 | EHEC | ARC77_10995 | 100 | 100 |
| *Escherichia coli* O157:H7 JEONG-1266 | EHEC | JEONG1266_0056 | 100 | 100 |
| *Escherichia coli* O157:H7 SS52 | EHEC | SS52_4096 | 100 | 100 |
| *Escherichia coli* O157:H7 SS17 | EHEC | SS17_3939 | 100 | 100 |
| *Escherichia coli* PA20 | EHEC | AU473_07155 | 100 | 100 |
| *Escherichia coli* SRCC1675 | EHEC | AR439_09590 | 100 | 100 |
| *Escherichia coli* O157:H7 WS402 | EHEC | AO055_18925 | 100 | 100 |
| *Escherichia coli* NADC5570-6564 | EHEC | BHW77_04450 | 100 | 100 |
| *Escherichia coli* NADC5570-6565 | EHEC | BHW76_04450 | 100 | 100 |
| *Escherichia coli* O145:H28 RM13516 | EHEC | ECRM13516_3651 | 100 | 100 |
| *Escherichia coli* O145:H28 RM13514 | EHEC | ECRM13514_3806 | 100 | 100 |
| *Escherichia coli* O145:H28 RM12581 | EHEC | ECRM12581_18735 | 100 | 100 |
| *Escherichia coli* O145:H28 RM12761 | EHEC | ECRM12761_17835 | 100 | 100 |
| *Escherichia coli* O26:H11 11368 | EHEC | ECO26_4015 | 99.3 | 98.6 |
| *Escherichia coli* FORC028 | EHEC | FORC28_0910 | 99.3 | 99.3 |
| *Escherichia coli* O111:H- 11128 | EHEC | ECO111_3664 | 99.3 | 98.6 |
| *Escherichia coli* 268-78-1 | EHEC | AL551_20460 | 99.3 | 99.3 |
| *Escherichia coli* O103:H2 12009 | EHEC | ECO103_3503 | 99.3 | 98.6 |
| *Escherichia coli* O55:H7 RM12579 | EPEC | ECO55CA74_17075 | 100 | 100 |
| *Escherichia coli* 2013C-4465 | EPEC | A5955_05850 | 100 | 100 |
| *Escherichia coli* O55:H7 CB9615 | EPEC | G2583_3582 | 100 | 100 |
| *Escherichia coli* O127:H6 E2348/69 | EPEC | E2348C_3175 | 90.3 | 93.0 |
| *Escherichia coli* O104:H4 C227-11 | STEC | AAF13_01495 | 99.1 | 98.6 |
| *Escherichia coli* HUSEC2011 | STEC | HUS2011_3464 | 99.1 | 98.6 |
| *Escherichia coli* O104:H4 2009EL-2071 | STEC | O3O_20820 | 99.1 | 98.6 |
| *Escherichia coli* O104:H4 2011C-3493 | STEC | O3K_04830 | 99.1 | 98.6 |
| *Escherichia coli* O104:H4 2009EL-2050 | STEC | O3M_04875 | 99.1 | 98.6 |
| *Escherichia coli* CFSAN004176 | STEC | BCV59_19755 | 99.3 | 99.3 |
| *Escherichia coli* CFSAN004177 | STEC | CFSAN004177_06800 | 99.3 | 99.3 |
| *Escherichia coli* GB089 | STEC | CO57_18495 | 99.3 | 99.3 |
| *Escherichia coli* 09-00049 | STEC | GJ12_18830 | 99.3 | 99.3 |
| *Escherichia coli* 2011C-3911 | STEC | A5956_05235 | 99.3 | 99.3 |
| *Escherichia coli* 2012C-4227 | STEC | AKK22_20645 | 99.3 | 99.3 |
| *Escherichia coli* 55989 | STEC | EC55989_3216 | 99.1 | 97.9 |
| *Escherichia coli* SEC470 | STEC | AWH59_19810 | 99.1 | 98.6 |
| *Escherichia coli* RM9387 | STEC | HW42_19365 | 99.3 | 98.6 |
| *Escherichia coli* O139:H28 E24377A | STEC | EcE24377A_3258 | 99.3 | 98.6 |
| *Escherichia coli* SE15 | STEC | ECSF_2720 | 91.0 | 93.0 |
| *Escherichia coli* CFSAN029787 | EIEC | AA102_06570 | 99.3 | 99.3 |
| *Escherichia coli* 94-3024 | EAEC | HW43_19095 | 99.1 | 98.6 |
| *Escherichia coli* LF82 | AIEC | LF82_454 | 90.3 | 93.0 |
| *Escherichia coli* O83:H1 NRG-857C | AIEC | NRG857_14355 | 90.3 | 93.0 |
| *Escherichia coli* UM146 | AIEC | UM146_01905 | 90.7 | 93.0 |
| *Escherichia coli* CI5 | UPEC | VK74_20845 | 99.3 | 99.3 |
| *Escherichia coli* O25b:H4 | UPEC | WLH_01974 | 91.0 | 93.7 |
| *Escherichia coli* NA114 | UPEC | ECNA114_2968 | 91.0 | 93.0 |
| *Escherichia coli* CD306 | UPEC | AVR67_16410 | 91.0 | 93.0 |
| *Escherichia coli* MNCRE44 | UPEC | SY51_16420 | 91.0 | 93.0 |
| *Escherichia coli* JJ1887 | UPEC | AX202_16545 | 91.0 | 93.0 |
| *Escherichia coli* JJ1886 | UPEC | P423_16035 | 91.0 | 93.0 |
| *Escherichia coli* JJ2434 | UPEC | AVR68_16400 | 91.0 | 92.3 |
| *Escherichia coli* O25b:H4 ST131-EC958 | UPEC | EC958_3207 | 91.0 | 93.0 |
| *Escherichia coli* uk-P46212 | UPEC | AUO99_12450 | 91.0 | 93.0 |
| *Escherichia coli* ECOL732 | UPEC | A4X18_13855 | 91.0 | 93.0 |
| *Escherichia coli* ZH193 | UPEC | AVR69_16245 | 91.0 | 93.0 |
| *Escherichia coli* VR50 | UPEC | ECVR50_3173 | 90.7 | 93.0 |
| *Escherichia coli* UT189 | UPEC | UTI89_C3311 | 90.7 | 93.0 |
| *Escherichia coli* K-15KW01 | UPEC | BA058_07700 | 90.6 | 93.0 |
| *Escherichia coli* UPEC 26-1 | UPEC | BB344_23515 | 90.0 | 92.9 |
| *Escherichia coli* cloneD i14 | UPEC | i14_3227 | 90.0 | 92.9 |
| *Escherichia coli* cloneD i2 | UPEC | i02_3227 | 90.0 | 92.9 |
| *Escherichia coli* 536 | UPEC | ECP_2917 | 90.0 | 92.9 |
| *Escherichia coli* CFT073 | UPEC | c3506 | 90.0 | 92.9 |
| *Escherichia coli* O7:K1 CE10 | NMEC | CE10_3364 | 90.7 | 93.0 |
| *Escherichia coli* IAI39 | NMEC | ECIAI39_3342 | 90.7 | 93.0 |
| *Escherichia coli* RS218 | NMEC | W817_16555 | 90.7 | 93.0 |
| *Escherichia coli* MVAST0167 | ExpEC | AVR75_14755 | 91.0 | 93.0 |
| *Escherichia coli* G749 | ExpEC | AVR74_15700 | 91.0 | 93.0 |
| *Escherichia coli* JJ1897 | ExpEC | AVR73_16930 | 91.0 | 93.0 |
| *Escherichia coli* H1827-12 | ExpEC | AB847_19110 | 90.7 | 93.0 |
| *Escherichia coli* PPECC42 | ExpEC | - | 90.7 | 93.0 |
| *Escherichia coli* SF-173 | ExpEC | AN205_04280 | 90.7 | 93.0 |
| *Escherichia coli* SF-166 | ExpEC | AN204_04665 | 90.7 | 93.0 |
| *Escherichia coli* SF-468 | ExpEC | AN206_04735 | 90.7 | 93.0 |
| *Escherichia coli* SF-088 | ExpEC | AN203_04580 | 90.7 | 93.0 |
| *Escherichia coli* PMV-1 | ExpEC | ECOPMV1_03197 | 90.7 | 93.0 |
| *Escherichia coli* PCN033 | ExpEC | PPECC33_03182 | 90.7 | 93.0 |
| *Escherichia coli* UMN026 | ExpEC | ECUMN_3273 | 90.7 | 93.0 |
| *Escherichia coli* S88 | ExpEC | ECS88_3204 | 90.7 | 93.0 |
| *Escherichia coli* IHE3034 | ExpEC | ECOK1_3311 | 90.7 | 93.0 |
| *Escherichia coli* ST648 | ExpEC | FH07_19915 | 90.0 | 91.6 |
| *Escherichia coli* ZH063 | ExpEC | AVR76_16145 | 90.0 | 92.9 |
| *Escherichia coli* SaT040 | ExpEC | AVR72_16430 | 89.7 | 92.9 |
| *Escherichia coli* ACN002 | APEC | ACN002_2969 | 99.1 | 98.6 |
| *Escherichia coli* ACN001 | APEC | J444_3110 | 99.1 | 98.6 |
| *Escherichia coli* 1 | APEC | AC789_1c32630 | 99.1 | 98.6 |
| *Escherichia coli* APEC O78 | APEC | APECO78_18355 | 99.1 | 98.6 |
| *Escherichia coli* APEC-IMT5155 | APEC | L282_1124 | 90.7 | 93.0 |
| *Escherichia coli* SMS-3-5 | APEC | EcSMS35_3065 | 90.7 | 93.0 |
| *Escherichia coli* APEC O1 | APEC | APECO1_3605 | 90.7 | 93.0 |
| *Escherichia coli* 2 | Undefined | A8V37_14825 | 99.3 | 99.3 |
| *Escherichia coli* S51 | Undefined | A9K64_04235 | 99.3 | 99.3 |
| *Escherichia coli* ECC-1470 | Undefined | E1470_c30450 | 99.3 | 98.6 |
| *Escherichia coli* B7A | Undefined | L960_4031c | 99.3 | 98.6 |
| *Escherichia coli* IAI1 | Undefined | ECIAI1_3048 | 99.3 | 98.6 |
| *Escherichia coli* O177:H21 | Undefined | BB405_17035 | 99.1 | 98.6 |
| *Escherichia coli* 210205630 | Undefined | A9C00_09840 | 99.1 | 98.6 |
| *Escherichia coli* YD786 | Undefined | ASE18_13145 | 99.1 | 98.6 |
| *Escherichia coli* LY180 | Undefined | LY180_15070 | 99.1 | 98.6 |
| *Escherichia coli* W1 | Undefined | WFL_15540 | 99.1 | 98.6 |
| *Escherichia coli* KO11FL | Undefined | KO11_08130 | 99.1 | 98.6 |
| *Escherichia coli* W | Undefined | ECW_m3183 | 99.1 | 98.6 |
| *Escherichia coli* KO11 | Undefined | EKO11_0803 | 99.1 | 98.6 |
| *Escherichia coli* SE11 | Undefined | ECSE_3192 | 99.1 | 98.6 |
| *Escherichia coli* MRE600 | Undefined | AWB62_03490 | 98.6 | 97.2 |
| *Escherichia coli* Eco889 | Undefined | WM90_23110 | 91.0 | 93.7 |
| *Escherichia coli* Ecol448 | Undefined | A4R37_13305 | 91.0 | 93.7 |
| *Escherichia coli* Ecol745 | Undefined | A4R38_07910 | 91.0 | 93.7 |
| *Escherichia coli* Ecol743 | Undefined | A4R39_08990 | 91.0 | 93.7 |
| *Escherichia coli* 3 | Undefined | NCTC86EC_03329 | 90.7 | 93.0 |
| *Escherichia coli* FORC031 | Undefined | FORC31_0949 | 90.7 | 93.0 |
| *Escherichia coli* FAP1 | Undefined | LI75_17850 | 90.7 | 93.0 |
| *Escherichia coli* ATCC-25922 | Undefined | DR76_1698 | 90.3 | 93.0 |
| *Escherichia coli* ED1a | Undefined | ECED1_3384 | 90.3 | 92.3 |
| *Escherichia coli* NGF1 | Undefined | A9Z04_07215 | 90.2 | 93.0 |
| *Escherichia coli* 06-00048 | Undefined | GJ11_18870 | 89.8 | 92.9 |
| *Escherichia coli* Nissle-1917 | Undefined | ECOLIN_15755 | 90.0 | 92.9 |
| *Escherichia coli* ABU83972 | Undefined | ECABU_c32080 | 89.7 | 92.2 |
| *Escherichia coli* ECUNIH2 | Undefined | WM48_15775 | 89.6 | 90.1 |

^*^, EHEC, Enterohemorrhagic *E. coli*; EPEC, Enteropathogenic *E. coli*; STEC, Shiga toxin-producing *E. coli*; EAEC, Enteroaggregative *E. coli*; EIEC, Enteroinvasive *E. coli*; AIEC, Adherent-invasive *E. coli*; UPEC, Uropathogenic *E. coli*; NMEC, Neonatal-meningitis-associated *E. coli*; ExpEC, Extraintestinal pathogenic *E. coli*; APEC, Avian pathogenic *E. coli*; Undefined, undefined pathotypes.
