# Supplementary material for: Magnesium Sensing Regulates Intestinal Colonization of Enterohemorrhagic Escherichia coli O157:H7
Source: mBio. 2020 Nov 10;11(6):e02470-20. doi: 10.1128/mBio.02470-20 (PMC7667037; doi:10.1128/mBio.02470-20)
Supplement: TABLE S3 [file mBio.02470-20-st003.docx]

**Table S3. Strains and plasmids used in this study**

| **Strains** | **Genotype or description** | **Source or reference** |
| --- | --- | --- |
| EHEC O157 | Wild-type EHEC O157:H7 EDL933 | ATCC* |
| ΔOI-119 | EHEC O157 ΔOI-119 | This work |
| Δ*lmiA* | EHEC O157 Δ*lmiA* | This work |
| Δ*z4268* | EHEC O157 *z4268*::*kan* | This work |
| Δ*z4269* | EHEC O157 *z4269*::*kan* | This work |
| Δ*z4270* | EHEC O157 *z4270*::*kan* | This work |
| Δ*z4271* | EHEC O157 *z4271*::*kan* | This work |
| Δ*ler* | EHEC O157 Δ*ler* | This work |
| Δ*ler*Δ*lmiA* | EHEC O157 Δ*lmiA* Δ*ler* | This work |
| Δ*phoQ* | EHEC O157 Δ*phoQ* | This work |
| Δ*phoP* | EHEC O157 Δ*phoP* | This work |
| Δ*lmiA*Δ*phoQ* | EHEC O157 Δ*lmiA* Δ*phoQ* | This work |
| Δ*lmiA*Δ*phoP* | EHEC O157 Δ*lmiA* Δ*phoP* | This work |
| Δ*mgrA* | EHEC O157Δ*mgrA* | This work |
| Δ*grlA* | EHEC O157Δ*grlA* | This work |
| G4955 | Wild-type EHEC O26:H11 | Gansheroff LJ, 1999 |
| G4955Δ*lmiA* | G4955 *lmiA* orthologues::*kan* | This work |
| G1327 | Wild-type EHEC O103:H2 | BfR^†^ |
| G1327Δ*lmiA* | G1327 *lmiA* orthologues::*kan* | This work |
| G1345 | Wild-type EHEC O145:H28 | BfR |
| G1345Δ*lmiA* | G1345 *lmiA* orthologues::*kan* | This work |
| G4951 | Wild-type EHEC O111:H- | Lab collection |
| G4951Δ*lmiA* | G4951 *lmiA* orthologues::*kan* | This work |
| G2583 | Wild-type EPEC O55:H7 CB9615 | BfR |
| G4951Δ*lmiA* | G2583 *lmiA* orthologues::*kan* | This work |
| G3109 | Wild-type EPEC O127:H- | Lab collection |
| G4951Δ*lmiA* | G3109 *lmiA* orthologues::*kan* | This work |
| G1485 | Wild-type EPEC O145:H34 | BfR |
| G4951Δ*lmiA* | G1485 *lmiA* orthologues::*kan* | This work |
| ΔOI-119 (p*lmiA*) | EHEC O157 ΔOI-119 with p*lmiA* | This work |
| Δ*lmiA* (p*lmiA*) | EHEC O157 Δ*lmiA* with p*lmiA* | This work |
| Δ*ler* (p*ler*) | EHEC O157 Δ*ler* with p*ler* | This work |
| Δ*phoQ* (p*phoQ*) | EHEC O157 Δ*phoQ* with p*phoQ* | This work |
| Δ*phoP* (p*phoP*) | EHEC O157 Δ*phoP* with p*phoP* | This work |
| Δ*phoP*(p*trc-lmiA*) | EHEC O157 Δ*phoP* with p*trc-lmiA* | This work |
| Δ*lmiA*Δ*phoP* (p*lmiA*) | EHEC O157 Δ*lmiA* Δ*phoP* with p*lmiA* | This work |
| Δ*lmiA*Δ*phoP* (p*trc-lmiA*) | EHEC O157 Δ*lmiA* Δ*phoP* with p*trc-lmiA* | This work |
| Δ*lmiA*Δ*phoP* (p*phoP*) | EHEC O157 Δ*lmiA* Δ*phoP* with p*phoP* | This work |
| BL21 | F- *ompT* *hsdSB (rB-, mB-) gal dcm* (DE3) | Invitrogen |
| BL21-pET-*lmiA* | BL21 with pET-*lmiA* | This work |
| BL21-pET-*phoP* | BL21 with pET-*phoP* | This work |
| Δ*lmiA*-*lmiA*-FLAG | EHEC O157 Δ*lmiA* with pTR-*lmiA*-3×FLAG | This work |
| Δ*phoP*-*phoP*-FLAG | EHEC O157 Δ*phoP* with pTR-*phoP*-3×FLAG | This work |
| O157WT-*eae*-FLAG | EHEC O157 WT, intimin tagged with 3×FLAG | This work |
| Δ*lmiA*-*eae*-FLAG | EHEC O157 Δ*lmiA*, intimin tagged with 3×FLAG | This work |
| Δ*lmiA* (p*lmiA*)-*eae*-FLAG | EHEC O157 Δ*lmiA* with p*lmiA*, intimin tagged with 3×FLAG | This work |
| O157WT-*tir*-FLAG | EHEC O157 WT, Tir tagged with 3×FLAG | This work |
| Δ*lmiA*-*tir*-FLAG | EHEC O157 Δ*lmiA*, Tir tagged with 3×FLAG | This work |
| Δ*lmiA* (p*lmiA*)-*tir*-FLAG | EHEC O157 Δ*lmiA* with p*lmiA*, Tir tagged with 3×FLAG | This work |
| **Plasmids** | | |
| pKD46 | Red recombination plasmid, ApR | Datsenko KA, 2000 |
| pKD3 | Containing a chloramphenicol resistance cassette and the flipase recognition sites, CmR | Datsenko KA, 2000 |
| pKD4 | Containing a kanamycin resistance cassette and the flipase recognition sites, KmR | Datsenko KA, 2000 |
| pCP20 | FLP expression plasmid, ApR, CmR | Datsenko KA, 2000 |
| pACYC184 | Low copy expression vector, CmR, TcR | Chang AC, 1978 |
| pTRC99A | Expression vector, ApR | Amann E, 1988 |
| pET-28a | T7 expression vector, KmR | Novagen |
| p*lmiA* | pACYC184 carrying *lmiA*, including its promoter region, CmR | This work |
| p*trc-lmiA* | pTRC99A carrying *lmiA*, under control of the IPTG-inducible *trc* promoter | This work |
| p*ler* | pACYC184 carrying *ler*, including its promoter region, CmR | This work |
| p*phoQ* | pACYC184 carrying *phoQ*, including *phoP* promoter region, CmR | This work |
| p*phoP* | pACYC184 carrying *phoP*, including its promoter region, CmR | This work |
| pET-*lmiA* | pET-28a carrying *lmiA*, KmR | This work |
| pET-*phoP* | pET-28a carrying *phoP*, KmR | This work |
| pTR-*lmiA*-3×FLAG | pTRC99A carrying *lmiA*-3×FLAG, ApR | This work |
| pTR-*phoP*-3×FLAG | pTRC99A carrying *phoP*-3×FLAG, ApR | This work |

^*^, ATCC, American Type Culture Collection, Manassas, Virginia, USA. ^†^, BfR, Beutin Federal institute for Risk Assessment National Reference Laboratory for *Escherichia coli,* Berlin, Germany.
