# Supplementary material for: Magnesium Sensing Regulates Intestinal Colonization of Enterohemorrhagic Escherichia coli O157:H7
Source: mBio. 2020 Nov 10;11(6):e02470-20. doi: 10.1128/mBio.02470-20 (PMC7667037; doi:10.1128/mBio.02470-20)
Supplement: TABLE S4 [file mBio.02470-20-st004.docx]

**Table S4. Primers used in this study (5'-3')**

| Primers for gene mutation | | |
| --- | --- | --- |
| OI-119 | F | GAAAACAATTATCTGACTTACGTCACGTTTTCAGCCCCGTGTAGGCTGGAGCTGCTTCG |
| OI-119 | R | TATGACCGTACCGACGGGCCGAATGTTCGCCGGGTGCTGCATATGAATATCCTCCTTAG |
| *lmiA* | F-1 | ATATTATCCGTAATACGTCCGAGTTTCGGAGTACTCCAGTGTAGGCTGGAGCTGCTTCG |
| *lmiA* | F-2 | ATATTATCCGCAATACGTCCGAGTTTCGGAGTACTCCAGTGTAGGCTGGAGCTGCTTC |
| *lmiA* | R-1 | AAGTGGCTACGCGCCATAAGAAAACCTCTCTGAAAGTAGCATATGAATATCCTCCTTAG |
| *lmiA* | R-2 | AAGTGGCTACGCGCCATAAGAAAACCTCTTTGAAAGTAGATGGGAATTAGCCATGGTCC |
| *lmiA* | R-3 | AAGTGGCGACGCGCCATAAGAAAACCTCTCTGAAAATAGATGGGAATTAGCCATGGTCC |
| *z4268* | F | TTCCCCCGTTAAATAACTACTTTCAGAGAGGTTTTCTTGTGTAGGCTGGAGCTGCTTCG |
| *z4268* | R | GCCAATGCCCATAGTGTTAATGAGGTAAATGGATGCATATGGGAATTAGCCATGGTCC |
| *z4269* | F | CCACGCGCCATTTTCCGGCAATGGCTGCCGTGCGCTAAGTGTAGGCTGGAGCTGCTTCG |
| *z4269* | R | TGTGTCGGGCAATAACGGAACTGTTCTAACGTAACCATATGGGAATTAGCCATGGTCC |
| *z4270* | F | TGCTGACAGAATTCGGAGCGTGGATATGGTTACGTTAGGTGTAGGCTGGAGCTGCTTCG |
| *z4270* | R | AACATAGTGGCTGGAGCGCTCCGTTTTGCAATTGCCACATGGGAATTAGCCATGGTCC |
| *z4271* | F | CGTCATCGCACACCTTTCCTGAACTACTGCCAGCAAGCGTGTAGGCTGGAGCTGCTTCG |
| *z4271* | R | AGTCTGCCTGCAAATTTGACCTTAATGATGACCGATGGGAATTAGCCATGGTCC |
| *ler* | F | TTCCAGCTCAGTTATCGTTATCATTTAATTATTTCATGGTGTAGGCTGGAGCTGCTTCG |
| *ler* | R | CTTCCTGATAAGGTCGCTAATAGCTTAAAATATTAAAGCCATATGAATATCCTCCTTAG |
| *phoQ* | F | GGGTACGTTTTCTGTTGGCAACGGCAGCGGTAGTACTGGTGTAGGCTGGAGCTGCTTCG |
| *phoQ* | R | AATCAATGGGTTGCCTTGCTGTGGTGATAAAAGGACATATCATATGAATATCCTCCTTAG |
| *phoP* | F | TTACACTATTTTAATAATTAAGACAGGGAGAAATAAAAGTGTAGGCTGGAGCTGCTTCG |
| *phoP* | R | GCAGCGAGAGCGGGAAAAAAAGACGCAGTAATTTTTTCACATATGAATATCCTCCTTAG |
| Primers for mutant verification | | |
| OI-119 | F | AAAGCACGAACCACATTACG |
| OI-119 | R | TTCAAACACGCTCAACCAAT |
| *lmiA* | F | TTGCCAGACAGAGTAATCCA |
| *lmiA* | R-1 | CCGCTGACAACGACTTTT |
| *lmiA* | R-2 | CCGCTAACAACGACTTTT |
| *z4268* | F | CGCTTTCTGGTTACGACG |
| *z4268* | R | GCTCCCAGCGAGAACATC |
| *z4269* | F | ACCCTGCCAAAAGTCGTT |
| *z4269* | R | GTCCCCGTGCCTGTTATC |
| *z4270* | F | TCCACAACCGACGAACAA |
| *z4270* | R | GTTGGTTTTCCGCTTCCT |
| *z4271* | F | GCGTGGAAGAAGAAGTTGC |
| *z4271* | R | GTCGTGGAAGGGGATTGTA |
| *ler* | F | TTATTTCTTGTTTGGCTCAC |
| *ler* | R | ATTGTTGGTCCTTCCTGAT |
| *phoQ* | F | GCAAAGTGGTCAGCAAAGATTC |
| *phoQ* | R | CCCATATCGACATTAACGC |
| *phoP* | F | GAAGAGAAAGCCCGCCTGA |
| *phoP* | R | GGTAACTCGACATGCAACTTA |
| Primers for gene cloning | | |
| *lmiA* | F | CATGCCATGGCAATGATGACATTGCCCTACCC |
| *lmiA* | R | CGGGATCCTTTAACGGGGGAAATATGC |
| *lmiA* | R | CGGGATCCTTATTTAACGGGGGAAATATGC |
| *lmiA* | F | CCCAAGCTTCTGTCGCAAACTGCGACT |
| *lmiA* | R | CGGGATCCTTATTTAACGGGGGAAATA |
| *ler* | F | CCCAAGCTTTAAGGTGGTTGTTTGATGAAAT |
| *ler* | R | CGGGATCCTTTAAATATTTTTCAGCGGTATTAT |
| *phoQ* | F-1 | CCCAAGCTTCGGCTAACTACATTGGTCG |
| *phoQ* | R-1 | CCGTAGGCAAGCGAAAGCACTTTTATTTCTCCCTGTCTTAA |
| *phoQ* | F-2 | TTAAGACAGGGAGAAATAAAAGTGCTTTCGCTTGCCTACGG |
| *phoQ* | R-2 | CGGGATCCTTATTCATCTTTCGGCGTAGA |
| *phoP* | F | CATGCCATGGAAATGCGCGTACTGGTTGT |
| *phoP* | R | CGGGATCCTTTTCATCAGCGCAATTCG |
| *phoP* | F | CCCAAGCTTCGGCTAACTACATTGGTCG |
| *phoP* | R | CGGGATCCTTTTCATCAGCGCAATTCG |
| Primers for *lmiA*-3×FLAG and *phoP*-3×FLAG | | |
| *lmiA*-3F | F | CATGCCATGGCAATGATGACATTGCCCTACCC |
| *lmiA*-3F | R | CGGGATCCTTACTATTTATCGTCGTCATCTTTGTAGTCGATATCATGATCTTTATAATCACCGTCATGGTCTTTGTAGTCTTTAACGGGGGAAATATGCA |
| *phoP*-3F | F | CATGCCATGGAAATGCGCGTACTGGTTGT |
| *phoP* -3F | R | CGGGATCCTTACTATTTATCGTCGTCATCTTTGTAGTCGATATCATGATCTTTATAATCACCGTCATGGTCTTTGTAGTCGCGCAATTCGAACAAATAG |
| Primers for qRT-PCR | | |
| *rrsH* | F | GAAAGCGTGGGGAGCAAAC |
| *rrsH* | R | ACATGCTCCACCGCTTGTG |
| *eae* | F | GACGGTAGTTCACTGGACTTCTT |
| *eae* | R | TCGCCACCAATACCTAAACG |
| *tir* | F | AAAGCAGCAGGCGAAGAGG |
| *tir* | R | TCGGCACCTGCGAATCAT |
| *ler* | F | CAGGAAGCAAAGCGACTG |
| *ler* | R | ACCAGGTCTGCCCTTCTT |
| *escT* | F | GCAATAGATGCGGCTGGAC |
| *escT* | R | TCGGCTTGTAATGGTAATATCTCG |
| *escC* | F | GACCAAAATGTTGTCGTCCCA |
| *escC* | R | AGGTTACCGCTTCGCTCG |
| *escN* | F | AGGTTTTCTTGTTGCCTTTTGA |
| *escN* | R | TCTCCATTGGTCTGCCTATGC |
| *espB* | F | AAAACTCCTCGGCAAGATGG |
| *espB* | R | AATAATCCCGCCAACCAAAG |
| *lmiA* | F | CGGGCAAGAAGGCACCA |
| *lmiA* | R | ATGACCGCCAAGCATCGT |
| Primers for EMSA | | |
| P_LEE1_ | F | TCCTGGGGATTCACTCGCTTG |
| P_LEE1_ | R | TCATAATAAATAATCTCCGC |
| P_LEE1_-1 | F | TCCTGGGGATTCACTCGCTTG |
| P_LEE1_-1 | R | GATATAGAAGAAAATCATCTCGATTGCATTTCCATTTAGTTAA |
| P_LEE1_-1 | F | TTAACTAAATGGAAATGCAATCGAGATGATTTTCTTCTATATC |
| P_LEE1_-1 | R | TCATAATAAATAATCTCCGC |
| P_LEE1_-2 | F | TCCTGGGGATTCACTCGCTTG |
| P_LEE1_-2 | R | GAAGAAAATCATCTCGTTAACACCATCAGTTAATAATTGCATTTCCATTTAGTTAA |
| P_LEE1_-2 | F | TAAATGGAAATGCAATTATTAACTGATGGTGTTAACGAGATGATTTTCTTCTATATC |
| P_LEE1_-2 | R | TCATAATAAATAATCTCCGC |
| P_LEE2/3_ | F | CATTACTGCACCAGAAGGAC |
| P_LEE2/3_ | R | AGATTCATCTGCAGGCTCTG |
| P_LEE4_ | F | CGCATCGCACCATTGAGAAG |
| P_LEE4_ | R | CATTAGCCATTGGAAACTCACG |
| P_LEE5_ | F | TAGTTTGCTTAATTGGTTTTCTTTGGC |
| P_LEE5_ | R | ACCAATAGGCATAAATATCTC |
| P*_lmiA_* | F | GTCCAGTCAAAGAAAACAA |
| P*_lmiA_* | R | AATATGGGTAGGGCAATG |
| P*_lmiA_*_-1_ | F | GTCCAGTCAAAGAAAACAATTATCTCAGCCCCGCCCCTGTGGAAT |
| P*_lmiA_*_-1_ | R | AATATGGGTAGGGCAATG |
| P*_lmiA_*_-2_ | F | GTCCAGTCAAAGAAAACAATTATCTGACCCTCGTCACCAACTCAGCCCCGCCCCTGTGGAAT |
| P*_lmiA_*_-2_ | R | AATATGGGTAGGGCAATG |
| *rpoS* | F | CTTCCAGTGTTGCCGCT |
| *rpoS* | R | CCCGTACTATTCGTTTGCC |
| Primers for ChIP-qPCR | | |
| P_LEE1_ | F | GCTTGGTTTTTATTCTGTTTTATTTGT |
| P_LEE1_ | R | ACATCTATTTCATCAAACAACCACC |
| P_LEE2/3_ | F | ATTTCCCAATAATCTTAAAAACTCTTC |
| P_LEE2/3_ | R | AATCACTCCTGTCTTCTCATCCAC |
| P_LEE4_ | F | CCGATTACGCATCGCACC |
| P_LEE4_ | R | CATTAGCCATTGGAAACTCACG |
| P_LEE5_ | F | ATGTTCAGCCGTTTATCGACTAC |
| P_LEE5_ | R | GGGATTATGACCAAGATTACCAA |
| P*_lmiA_* | F | AGACAGAGTAATCCAGCAAATCG |
| P*_lmiA_* | R | TTGTGACGGCACAACGAAA |
| *rpoS* | F | GTTATCGCAGGGAGCCACA |
| *rpoS* | R | TTTTACCACCAGACGCAAGTTA |
